# Requirement of cAMP Signaling for Schwann Cell Differentiation Restricts the Onset of Myelination

**DOI:** 10.1371/journal.pone.0116948

**Published:** 2015-02-23

**Authors:** Ketty Bacallao, Paula V. Monje

**Affiliations:** 1 The Miami Project to Cure Paralysis, University of Miami Miller School of Medicine, Miami, Florida, United States of America; 2 Department of Neurological Surgery, University of Miami Miller School of Medicine, Miami, Florida, United States of America

## Abstract

Isolated Schwann cells (SCs) respond to cAMP elevation by adopting a differentiated post-mitotic state that exhibits high levels of Krox-20, a transcriptional enhancer of myelination, and mature SC markers such as the myelin lipid galactocerebroside (O1). To address how cAMP controls myelination, we performed a series of cell culture experiments which compared the differentiating responses of isolated and axon-related SCs to cAMP analogs and ascorbate, a known inducer of axon ensheathment, basal lamina formation and myelination. In axon-related SCs, cAMP induced the expression of Krox-20 and O1 without a concomitant increase in the expression of myelin basic protein (MBP) and without promoting axon ensheathment, collagen synthesis or basal lamina assembly. When cAMP was provided together with ascorbate, a dramatic enhancement of MBP expression occurred, indicating that cAMP primes SCs to form myelin only under conditions supportive of basal lamina formation. Experiments using a combination of cell permeable cAMP analogs and type-selective adenylyl cyclase (AC) agonists and antagonists revealed that selective transmembrane AC (tmAC) activation with forskolin was not sufficient for full SC differentiation and that the attainment of an O1 positive state also relied on the activity of the soluble AC (sAC), a bicarbonate sensor that is insensitive to forskolin and GPCR activation. Pharmacological and immunological evidence indicated that SCs expressed sAC and that sAC activity was required for morphological differentiation and the expression of myelin markers such as O1 and protein zero. To conclude, our data indicates that cAMP did not directly drive myelination but rather the transition into an O1 positive state, which is perhaps the most critical cAMP-dependent rate limiting step for the onset of myelination. The temporally restricted role of cAMP in inducing differentiation independently of basal lamina formation provides a clear example of the uncoupling of signals controlling differentiation and myelination in SCs.

## Introduction

The formation of a myelin sheath around axons is an exquisite example of the end result of a developmentally regulated highly coordinated cell differentiation process carried out exclusively by two specialized types of glial cells, the oligodendrocyte in the central nervous system and the Schwann cell (SC) in the peripheral nervous system (PNS). Early *in vitro* studies of SC myelination suggested that both the ensheathment of axons into one-to-one units and the assembly of a basal lamina on the abaxonal SC surface were required for the formation of a myelin sheath [1]. However, it was not up until recent years that experiments in animal models allowed the identification of the molecular signals that control myelination through axon contact- and basal lamina-dependent mechanisms, respectively. In particular, membrane-bound neuregulin 1-type III, an agonist of ErbB/HER receptors, and laminin, an agonist of integrin receptors, were shown to play a key instructive role in the regulation of peripheral myelination [2, 3].

It has also become apparent that the onset and progression of myelination depends on the counterbalancing effect of positive and negative transcriptional regulators which are in turn controlled by a multiplicity of signals emanating from the extracellular environment and the SCs themselves [4]. This balance is illustrated by the cross-antagonistic interplay of signals between Krox-20, a transcriptional enhancer and master regulator of peripheral myelination [5], and c-Jun, a member of the activating protein-1 family of transcription factors whose expression not only inhibits myelination but also induces myelin loss and SC dedifferentiation [6].

Available evidence has suggested that SCs require signaling from the ubiquitous second messenger cyclic adenosine monophosphate (cAMP) to initiate the myelination program [7]. This idea was supported at least in part by observations in isolated SCs which showed that cAMP elevation directly increases the ratio of Krox-20 to c-Jun expression [6]. Prolonged cAMP stimulation drives cell cycle exit and increases the expression of an array of proteins and lipids specific to the myelinating SC phenotype [8]. Intermediates of the cAMP signaling system such as protein kinase A (PKA) and exchange protein activated by cAMP (EPAC) have been reported to regulate the process of myelination *in vitro* [9–11] and *in vivo* [12, 13]. The recent discovery of Gpr126, a highly conserved orphan G protein-coupled receptor (GPCR) that signals through heterotrimeric G proteins and cAMP [14], has reinforced the idea that cAMP plays a role in developmental myelination [12, 15, 16]. In support of this notion, it has been shown that the lack of Krox-20 expression and myelin formation that results from deletion of Gpr126 is restored by treatment with forskolin, a potent agonist of transmembrane adenylyl cyclase (tmAC) activity, and PKA overexpression [12]. However, the first messengers that increase cAMP in differentiating SCs remain still elusive.

This study was carried out to better understand the role of cAMP on the expression of critical markers of the myelinating SC phenotype and the formation of myelin sheaths using cell culture systems of stepwise complexity. We began our study by investigating the temporal progression of phenotypic changes during cAMP-induced differentiation and its connection to myelination. First, to reveal changes in the expression of markers of differentiation, we performed a series of experiments using isolated purified SCs. Then, to validate these results and reveal changes in myelination, we performed experiments using SC-neuron cultures, as essential features of nerve development such as axon ensheathment, basal lamina formation and myelin sheath formation can only be recapitulated in a co-culture system that consists of primary SCs and primary neurons [17]. To understand the underlying mechanism of cAMP’s action on SC differentiation, we used a variety of pharmacological tools targeting selected upstream components of the AC/cAMP signaling system. In particular, we used agonists and antagonists able to discriminate between tmAC and soluble AC (sAC) activities, as these two different ACs increase cAMP through GPCR-dependent and GPCR-independent mechanisms, respectively. Experiments were designed so as to compare the cellular responses to cAMP elevation and inhibition in SC-only and SC-neuron cultures. In turn, co-culture experiments were performed in the absence and presence of ascorbate, a known essential factor for basal lamina assembly and myelin sheath formation *in vitro*. The comparison of the cellular responses to cAMP, ascorbate and their combination in isolated and axon-related SCs allowed us to conclude that cAMP played a role in SC differentiation rather than myelination and that its action occurred prior to and likely independently of the onset of myelin basic protein (MPB) expression and the formation of a basal lamina. We also provide pharmacological evidence indicating a potential role for cAMP derived from at least two different sources, namely the tmAC and sAC, at the onset of differentiation.

## Results

### cAMP induced a differentiated Krox-20 positive, O1 positive state but failed to drive the expression of MBP in isolated SCs

**Fig 1:**
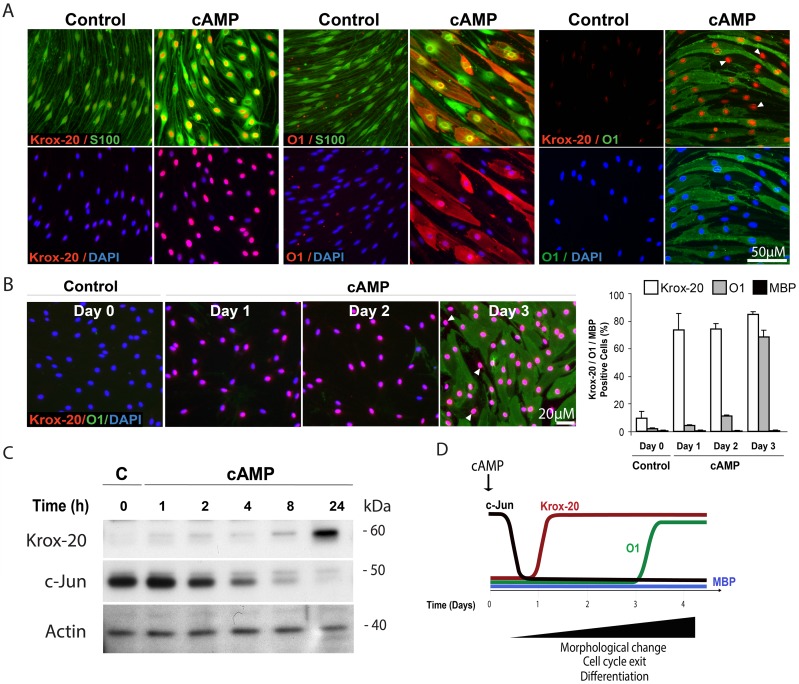
Induction of Krox-20 and O1 expression but not MBP in cAMP-treated isolated SCs. The figure depicts the temporal progression of phenotypic changes during cAMP-induced differentiation in isolated nerve-derived SC cultures. SCs were subjected to mitogen and serum starvation (Methods) prior to treatment with CPT-cAMP (250 μM) for 3 days unless otherwise indicated. The control condition in this and subsequent figures refers to cells that were incubated in the absence of cAMP-stimulating agents for the whole time course of the experiment. In panels B-C, the control (C) condition refers to cells that were fixed or lysed for analysis at the time of stimulation (day/hour zero). Cells were analyzed for the expression of c-Jun, Krox-20, O1 and MBP by immunofluorescence microscopy and western blot, as marked in the figure. In A, SCs were co-stained with the SC-specific marker S-100 to label all cells and reveal the changes in cell morphology that occur in response to prolonged CPT-cAMP stimulation. The schematic diagram (D) depicts the temporal course of changes during cAMP-induced differentiation as revealed by this and our previous time course studies [8]. Arrowheads in A-B point out to representative Krox-20 positive cells (nuclear localization) that fail to express O1. Cells that labeled positive for O1 and negative for Krox-20 were not found. Of note, staining with MBP antibodies did not detect a specific signal in either control or cAMP-treated cells (B, quantification shown on the right). All antibodies used are indicated in the figure; nuclei were labeled with DAPI (blue) in this and all subsequent figures.

Cultured SCs actively proliferate in the absence of neurons but remain almost indefinitely undifferentiated unless stimulated with high doses of cAMP analogs [7, 8, 18] or co-cultured with dorsal root ganglion (DRG) neurons under conditions supportive of myelination [17, 19]. In SCs growing in the absence of axons, the provision of membrane permeable analogs of cAMP to the culture medium induces a series of molecular and phenotypic changes associated with cell cycle exit and differentiation into a myelinating SC-like state [8, 20]. As shown in Fig. 1, SCs treated with the phosphodiesterase-resistant cAMP derivative CPT-cAMP for three days acquire the following typical features: (1) An increase in the expression of nuclear Krox-20 (Fig. 1A-C) along with markers typical of myelinating SCs, including the myelin lipid galactocerebroside (O1 labeling in Fig. 1A-B) and the myelin proteins P_0_ (protein zero) and MAG (myelin associated glycoprotein), (not shown); (2) A reduction of the expression of c-Jun (Fig. 1C) and markers typical of immature SCs such as GFAP, p75^NGFR^ and N-cadherin (not shown); and (3) A cell shape transformation that involves the loss of the spindle-shaped morphology typical of immature cells and attainment of an enlarged epithelial-like shape, as revealed by immunostaining with the SC-specific marker S100 (Fig. 1A). We have shown previously that prolonged and persistent stimulation with cAMP analogs is required to not only initiate but also maintain a state of differentiation, as SCs rapidly dedifferentiate and thereby lose the expression of myelination-associated markers upon the removal of the cAMP stimulus [20].

One important feature of the temporal progression of changes induced by CPT-cAMP was the requirement of at least three days of uninterrupted cAMP stimulation to observe an induction of the expression of mid-term myelin-specific markers such as O1 and MAG (Fig. 1B, shown only for O1). Nevertheless, time course studies revealed that 24 hours of stimulation were sufficient to shift the balance of Krox-20 to c-Jun expression (Fig. 1B-C). The temporal delay in the appearance of O1 with respect to the antagonistic changes in Krox-20 and c-Jun was consistent with the occurrence of a fraction of Krox-20 positive cells that did not express O1 (Fig. 1A-B). The proportion of Krox-20 positive, O1 negative cells typically ranged between 10 to 30% of total cells at 3 days post-stimulation (Fig. 1B) while cells expressing O1 and no nuclear Krox-20 were virtually absent in the cAMP-treated populations (Fig. 1A-B). A second important feature of the progression of changes induced by CPT-cAMP was the failure of cAMP-treated SCs to efficiently express MBP at the protein level (Fig. 1B) regardless of the time of exposure or the use of repeated additions of cAMP analogs (not shown). A schematic diagram summarizing the temporal course of phenotypic and molecular changes elicited by cAMP in isolated SCs is provided in Fig. 1D. Based on these results, we decided to rely on the expression of cell surface O1 to help us discriminate between differentiated, non-proliferative (O1 positive) and immature, proliferative (O1 negative) cells. For practical purposes, we have referred to the state of differentiation induced by cAMP as the O1 positive state.

### Ascorbate rather than cAMP elevation induced MBP expression in axon-associated SCs

The observation that cAMP-treated SCs were almost indefinitely arrested in an O1 positive, MBP negative state suggested the requirement of a different complement of signals to trigger the expression of MBP, the latter being a major constituent of the myelin sheath and one of the most widely used markers for the identification of myelinating cells. It is known that the addition of a source of ascorbate to the culture medium enables axon-contacting SCs to readily activate the expression of MBP and subsequently form a myelin sheath [17]. Available data indicate that ascorbate promotes myelination indirectly via stabilizing triple-helical collagen fibers and allowing SCs to assemble a basal lamina, which is a crucial prefatory step for the ensheathment of axons with cytoplasm and the wrapping of myelin membranes [17, 21, 22].

**Fig 2:**
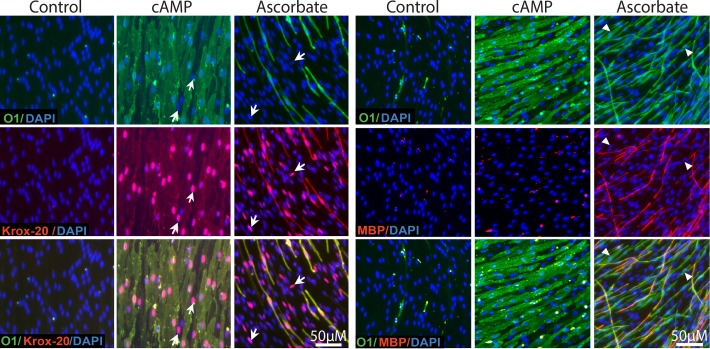
Induction of Krox-20 and O1 expression but not MBP in cAMP-treated SC-neuron cultures. SCs growing in co-culture with purified DRG neurons were established and stimulated as described in Methods. Co-cultures were left untreated (control), treated with CPT-cAMP (20 μM) or ascorbate (50 μg/ml), and analyzed by immunofluorescence microscopy 12 days after treatment initiation. A side-by-side analysis of Krox-20, O1 and MBP expression is shown. In this and all other experiments, specific treatments were provided every 3 days throughout the time course of the experiment. Representative areas of SC-neuron cultures where SCs maintain an association with DRG axons were selected to denote the quality of the changes in SC differentiation. Prolonged CPT-cAMP administration induced Krox-20 and O1 expression in the majority of the SCs but failed to induce myelination, as judged by the enlarged, non-compacted morphology of the O1 positive SCs and their lack of MBP expression. The arrows indicate representative Krox-20 positive, O1 negative SCs (left panels); the arrowheads indicate O1 positive, MBP negative SCs (right panels). Virtually no O1 negative, MBP positive cells were observed under this or any other culture condition.

Thus, we tested whether cAMP elevation could mimic the pro-myelinating action of ascorbate in axon-associated SCs by performing a detailed comparison of the differentiating effect of CPT-cAMP and L-ascorbic acid in a reconstituted co-culture system consisting only of SCs and DRG neurons. For this, the culture medium was supplemented with CPT-cAMP or ascorbate throughout the differentiation/myelination phase and the cultures were analyzed for the expression of markers of differentiation, including Krox-20, O1 and MBP (Fig. 2). Results indicated that treatment with CPT-cAMP was sufficient to effectively induce the expression of nuclear Krox-20 and cell surface O1 in axon-related SCs but was insufficient to induce the expression of MBP (Fig. 2). As expected, MBP expression was only observed under conditions of ascorbate supplementation. A microscopic analysis of MBP positive myelin segments in ascorbate-treated SC-neuron cultures further revealed a delayed expression of MBP with respect to O1. MBP/O1 co-localization studies indicated that only a proportion of the O1 positive cells concurrently exhibited MBP expression whereas cells expressing MBP in the absence of O1 were not found (Fig. 2).

**Fig 3:**
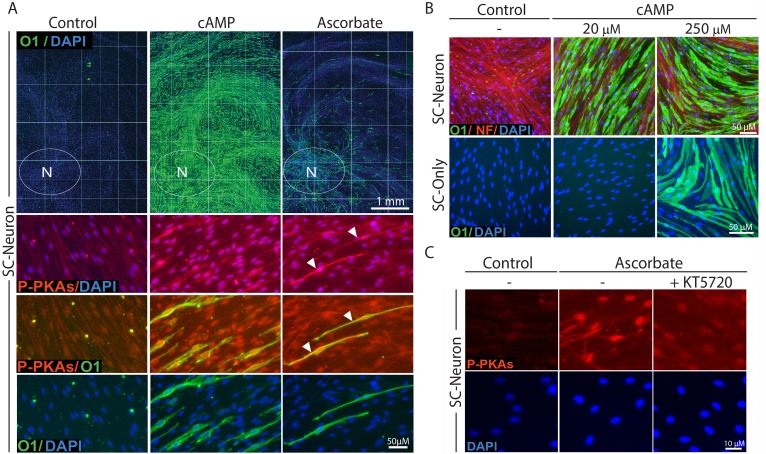
Induction of O1 expression in cAMP-treated SC-neuron cultures: dose dependency, spatial distribution and comparison to ascorbate’s effect. SCs growing alone (SC-only) or together with purified DRG neurons (SC-neuron) were treated with CPT-cAMP (20 μM unless otherwise specified) or ascorbate (50 μg/ml) and analyzed by immunofluorescence microscopy at 12 days (A, upper panels), 5 days (A, lower panels, and C) or 3 days (B) after treatment initiation. Representative cultures are shown at low (A, upper panels) and high magnification (A, lower panels, and B-C) to represent the magnitude and quality of the changes in O1 expression, respectively. Note that ascorbate induced the appearance of O1 positive cells only in a minor proportion of the SC population that was usually restricted to the axonal environment adjacent to the neuronal bodies (N, white ovals). On the contrary, cAMP induced O1 expression in the majority of the SCs regardless of their relative location within the axonal web. Images of double immunostaining with O1 and P-PKA substrate (P-PKAs) antibodies are shown to denote the effectiveness of cAMP and ascorbate treatment (A, lower panels). As a control for signal specificity, SC-neuron cultures were treated with ascorbate in the absence or presence of the PKA antagonist KT5720 (0.5 μM) prior to cell fixation and immunostaining with P-PKA substrate antibodies (C). The arrowheads in B point out to O1 positive cells that display high cytoplasmic P-PKA substrate labeling along the main axis of the cell. In this and all subsequent experiments, P-PKA substrate immunoreactivity is shown at 5 days post-stimulation because the signal intensity usually declines thereafter.

Several features of the SC-neuron cultures revealed a different mode of action of CPT-cAMP and ascorbate in regulating SC differentiation. Whereas ascorbate treatment induced O1 and MBP expression preferably in those SCs residing in the proximity of the neuronal bodies (as indicated by the area delimited by the white ovals in Fig. 3A), treatment with CPT-cAMP induced O1 expression throughout the axonal extension (i.e. without regard to the relative location of the SCs within the culture system) even when provided at low concentrations (Fig. 3A). Dose response experiments indicated that concentrations of CPT-cAMP that were 10 times lower than those required to induce differentiation in SC-only cultures were sufficient to drive optimal levels of Krox-20 and O1 expression in SC-neuron cultures (Fig. 3B, shown only for O1) possibly due to the influence of neuronal/axonal factors on SC differentiation.

To reveal the effectiveness of treatments inducing or inhibiting the intracellular levels of cAMP in SC cultures, we evaluated the expression of phosphorylated PKA-specific (P-PKA) substrates by immunofluorescence microscopy. We have previously shown by means of western blot and immunofluorescence microscopy that the expression of P-PKA substrates provides a reliable indication of the relative changes and subcellular distribution of cAMP/PKA activity in individual SCs [11]. As shown in Fig. 3A, treatment with CPT-cAMP and ascorbate induced high levels of nuclear P-PKA substrate expression in nearly all SCs. In particular, ascorbate-treated O1 positive cells were also identified by their strong cytoplasmic P-PKA substrate labeling (Fig. 3B), indicative of locally enhanced levels of cAMP in differentiating axon-related SCs. As a specificity control, we show that the immunodetection of P-PKA substrates in ascorbate-treated SC-neuron cultures was reduced to nearly basal levels in the presence of the PKA inhibitor KT5720 (Fig. 3C).

In spite of cAMP’s effectiveness to promote SC differentiation into an O1 positive state, persistent administration of CPT-cAMP did not mimic the action of ascorbate to enable axon-related SCs to enhance their expression of MBP.

### Ascorbate rather than cAMP enabled collagen type IV synthesis, basal lamina assembly, axon ensheathment and myelination in axon-associated SCs

**Fig 4:**
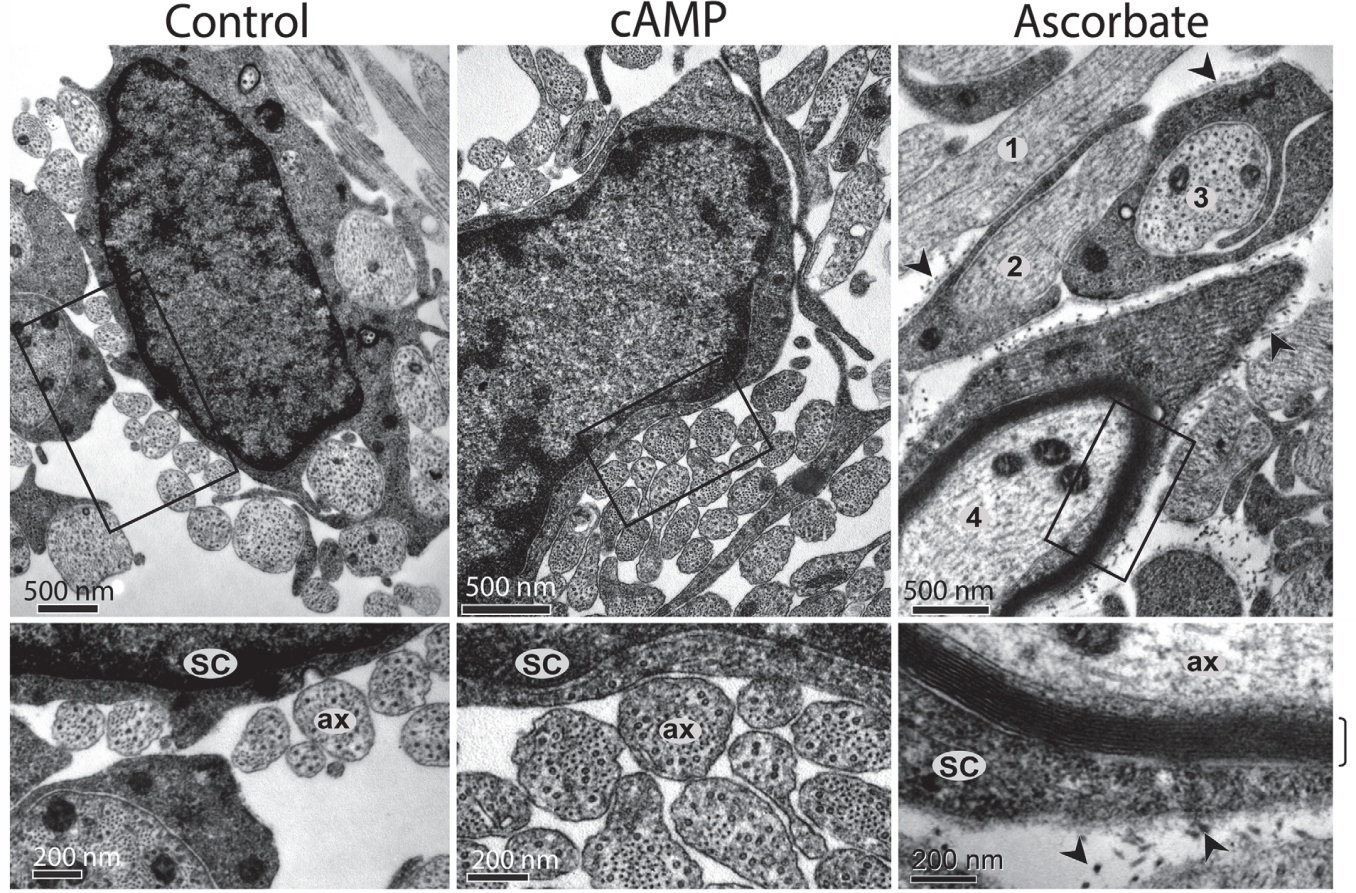
Absence of axon ensheathment, myelin and basal lamina in cAMP-treated SC-neuron cultures. Cells that were subjected to the same experimental treatments as in Fig. 2 were fixed and analyzed by TEM (Methods). Higher magnification images of selected areas (boxes) are shown to better resolve the interaction between SC processes (SC) and axons (ax). As opposed to ascorbate, CPT-cAMP treatment did not support the ensheathment of axons, the formation of a basal lamina, the deposition of extracellular collagen fibers (arrowheads) or the production of myelin membranes (bracket). Similar to the control condition (left panels), CPT-cAMP-treated SCs (cAMP) associated with multiple low diameter axons leaving many neurites deprived of direct contact with SC processes. The asynchronous responses in myelination induced by ascorbate are illustrated by the multiple ensheathment profiles shown in the upper right panel, as follows: (1) – No ensheathment; (2) Partial ensheathment; (3) Complete ensheathment, no myelin; (4) Complete ensheathment, myelin.

An interesting feature of cAMP-treated SCs was their arrest in a pre-myelinating O1 positive, MBP negative stage which could not be overcome by the availability of an axonal substrate or prolonged treatment with CPT-cAMP (Figs. 2–3). Despite the induced morphological changes, cAMP failed to promote the elongated bipolar phenotype characteristic of ensheathing SCs at their pre-myelinating or myelinating stage (Figs. 2 and 3A-B). Because the association of SCs and axons into one-to-one units is a pre-requisite for myelination, we performed a TEM analysis of SC-neuron cultures treated and non-treated with CPT-cAMP and ascorbate to investigate ultrastructural features of the SC-axon interaction. TEM results confirmed that CPT-cAMP treatment did not increase axon ensheathment or the formation of one-to-one units of SCs and axons (Fig. 4, middle panel). Of note, TEM images also revealed that CPT-cAMP-treated cultures did not exhibit the deposition of extracellular collagen fibers or the formation of a basal lamina (Fig. 4). Consistent with the lack of a basal lamina, CPT-cAMP-treated cultures showed no evidence on the existence of lamellar structures indicative of total or partial myelination (Fig. 4). At the TEM level, cAMP-treated cultures were essentially indistinguishable from control cultures maintained in the absence of ascorbate, which normally exhibit many DRG axons loosely attached to or even totally deprived of physical contact with SC processes (Fig. 4, left and middle panels). Interestingly, TEM analysis revealed that ascorbate induces myelination asynchronously within the SC population, as multiple stages of axon ensheathment and myelin synthesis are found at any given time during the myelination phase (Fig. 4, right panel).

Taken as a whole, these results suggest that cAMP elevation allowed SCs to differentiate into an O1 positive state without directly inducing MBP expression and basal lamina formation. These results not only revealed a delayed expression of MBP with respect to O1 but also a likely independent mechanism of control underlying O1 and MBP expression.

### cAMP potently increased MBP expression only under conditions supportive of axon ensheathment and basal lamina formation

**Fig 5:**
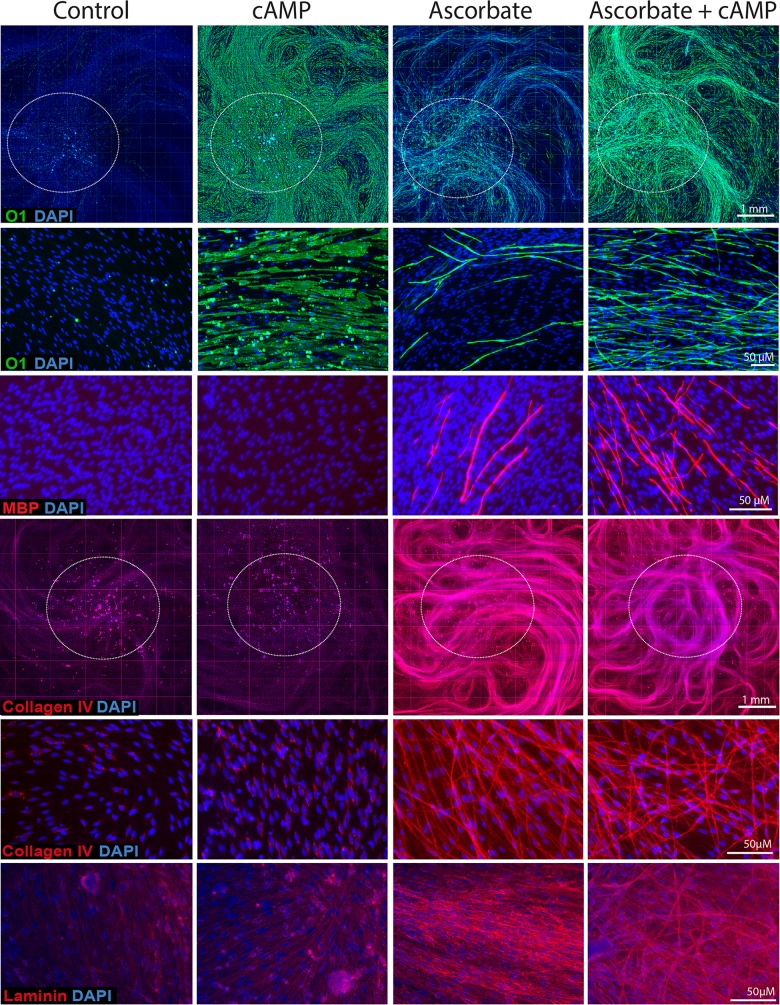
Expression of markers of differentiation and basal lamina in SC-neuron cultures treated with CPT-cAMP and ascorbate provided alone and in combination. SC-neuron cultures were obtained and stimulated essentially as described in Fig. 2A with the exception of the inclusion of a condition where CPT-cAMP (20 μM) was provided together with ascorbate (right panels). Cultures were stained with antibodies against O1, MPB, collagen type IV and laminin, as indicated. Low magnification composites of representative cultures stained with O1 (green staining) and collagen type IV (red staining) are shown to reveal changes over a large surface area. The region that contains the neuronal bodies is indicated by the white circles in each panel. Higher magnification (20x) images are shown for all markers. Note the widespread action of cAMP to increase O1 expression throughout the culture system regardless of the presence of ascorbate. Likewise, note the widespread action of ascorbate to increase collagen type IV expression regardless of the presence of cAMP. In ascorbate-free medium, some SCs exhibit cytoplasmic granules of collagen type IV. In ascorbate-containing medium, on the contrary, the expression of collagen type IV and laminin were mostly extracellular. Similar to collagen type IV and laminin, MBP expression was only detected in the presence of ascorbate. Contrary to collagen type IV and laminin, MBP expression was greatly enhanced by the addition of cAMP. Despite the widespread deposition of extracellular collagen type IV fibers by most SCs, only a proportion of these cells exhibited O1 and MPB expression.

To confirm the above results, we used immunofluorescence microscopy analysis to examine the expression of markers of differentiation (O1 and MBP) and basal lamina formation (collagen type IV and laminin) that are responsive to CPT-cAMP, ascorbate and their combination (Fig. 5). Because SC-neuron cultures display high complexity especially in regards to the spatial distribution of myelinating cells throughout the axonal network, we chose to present our fluorescence microscopy data in the following general format: (1) Low magnification images covering at least one fourth of the total surface of the well, as these enabled the visualization of the spatial distribution and relative intensity of the labeling; and (2) High magnification images of representative areas, as these enabled the visualization of the morphology of the cells and signal co-localization of multiple labels.

Results presented in Fig. 5 provided insight into the differential mode of action of CPT-cAMP and ascorbate in axon-related SCs. On one hand, CPT-cAMP alone promoted a widespread induction of O1 expression regardless of the presence of ascorbate but failed on its own to directly induce the expression of MBP and basal lamina constituents such as collagen type IV and laminin. On the other hand, cAMP greatly enhanced the expression of MBP when provided together with ascorbate without further increasing the expression of markers specific to the basal lamina. Dose response experiments suggested that the failure of CPT-cAMP to drive MBP expression was not improved if higher doses or different cell permeable analogs of cAMP were used (not shown). In the presence of ascorbate, high doses of CPT-cAMP (250 μM) allowed SCs to promptly acquired O1 expression while preventing the expression of MBP (S1 Fig.), possibly due to deleterious effects of high cAMP concentrations on the DRG neurons. It seems apparent that the widespread induction of O1 expression in SC-neuron cultures likely reflects the effect of cAMP on its own regardless of ascorbate administration. In turn, ascorbate treatment by itself enables maximal and extensive expression of extracellular matrix components regardless of cAMP administration.

**Fig 6:**
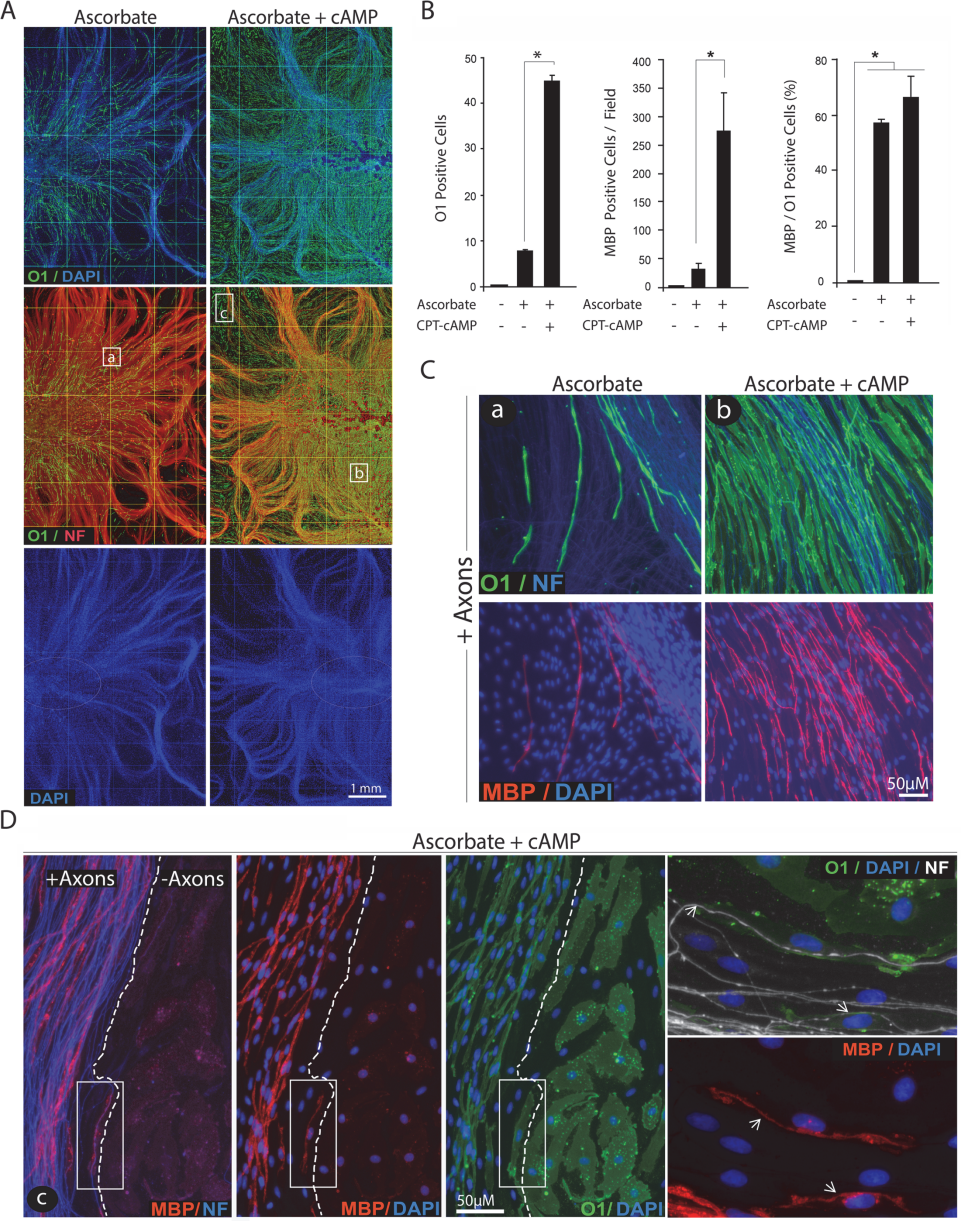
Induction of O1 and MBP expression by combined administration of cAMP and ascorbate: spatial distribution of O1 and MPB positive cells and dependency on axonal contact. Experimental conditions were identical to those of Fig. 5. In A, low magnification composites of representative cultures stained with O1 (green), neurofilament (red) and DAPI (blue) are shown to reveal the effect of the indicated treatments on O1 expression with respect to the location of the neuronal bodies (white ovals), the extension of the neurite substrate (neurofilament, NF) and the distribution of the SCs (DAPI). A quantitative analysis of O1 and MBP expression is provided in B (Methods). This analysis confirmed that cAMP enhanced the total number of O1 and MBP positive cells without concomitantly increasing the MBP/O1 ratio. Selected areas within the center (a, b) and periphery (c) of the axonal outgrowth are shown at higher magnification in C and D, respectively, to reveal details of the morphology of the cells, the relationship to axons and the co-localization of O1 (green) and MBP (red). Whereas O1 positive cells could be found throughout the culture system, MBP positive cells were found exclusively within the axonal network (C, D). Images taken at the frontier of the axonal outgrowth (D) revealed that MBP rather than O1 expression was restricted to those SCs that established a direct contact with the axons, as revealed by neurofilament staining (dotted lines). The boxed area in panel D was enlarged to better resolve the distribution of the MBP staining with respect to the position of the axons (NF, arrows), the plasma membrane (O1) and the nuclei of the cells (DAPI). In these and all subsequent graphs, bar heights are means of triplicate determinations; error bars represent S.D, and * represents statistical significance for p < 0.05.

It was striking to observe that in the presence of combined ascorbate and cAMP not only the local density but also the distribution of O1 positive, MBP positive myelin segments within the axonal substrate was dramatically increased (Figs. 5 and 6A-B) without noticeably augmenting the MBP/O1 ratio (Fig. 6B) or the total number of SCs (Fig. 6A, DAPI channel). The unchanged relationship between MBP and O1 positive cells further suggests that cAMP’s effects on myelination occur through a mechanism that directly tackles O1 rather than MBP expression. Indeed, a close examination of cAMP-treated co-cultures at the edge of the axonal outgrowth revealed that the expression of MBP but not O1 was restricted to those SCs that established a direct physical interaction with live axons (Fig. 6D). Similar to SCs growing in isolation from neurons, SCs outside of the axonal web effectively responded to cAMP treatment by undergoing a morphological transformation and enhancing O1 expression in the absence of MBP. This result provides a clear indication that direct contact with axons rather than cAMP signaling is the limiting factor for effective MBP expression.

Taken together, these results suggest that cAMP neither mimics nor replaces the action of live axons to allow MBP expression in SCs. It seems apparent that cAMP and ascorbate do not overlap in their mode of action toward inducing the myelinating phenotype but are required for independently regulated steps as SCs differentiate into an O1 positive state, a cAMP-dependent event, and then express MBP and form myelin, an ascorbate-dependent event. Whereas the differentiating effect of cAMP seems to be direct, the effect of ascorbate is most likely indirect and reflects its well-demonstrated action on allowing basal lamina assembly and axon ensheathment. The functional uncoupling between the O1 and the MBP positive states of differentiation represents an invaluable opportunity to mechanistically dissect the regulation of two critical but still independent stages limiting the onset of myelination.

### The activity of the tmAC was required for myelination in SC-neuron cultures

**Fig 7:**
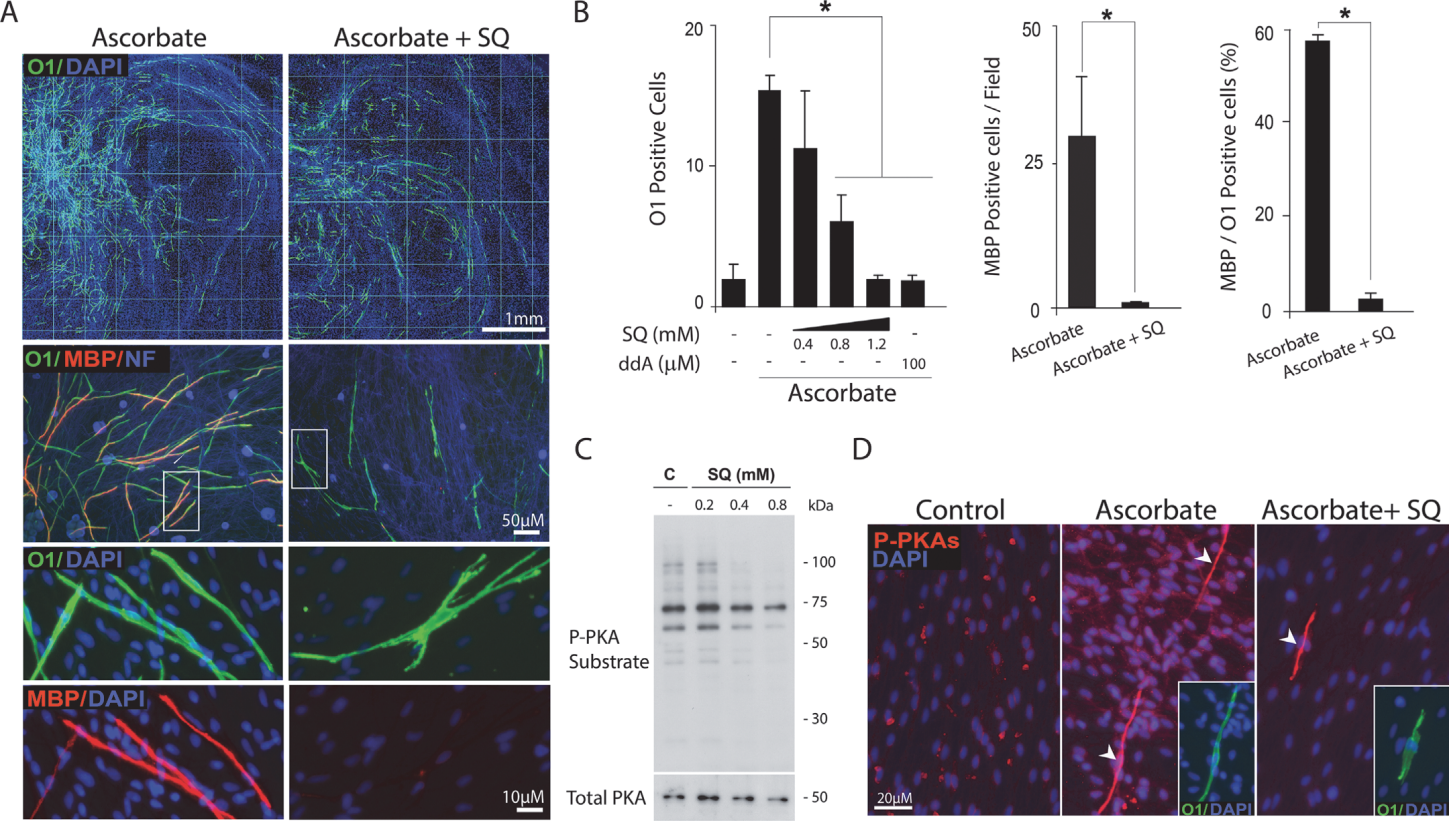
Reduction of O1 and MBP expression by pharmacological inhibition of the tmAC. SC-neuron cultures were induced to produce myelin by treatment with ascorbate in the absence (Control) or presence of SQ22536 (SQ), which was provided at 0.8 μM unless otherwise indicated. Treatment was carried out for 12 days (A-B) and 5 days (D), respectively. Cultures were analyzed for their expression of O1 and MBP (A-B) or O1 and P-PKA-specific substrates (D) by immunofluorescence microscopy. A quantification of O1 and MBP expression is provided in B, which also includes a condition where mtAC activity was inhibited by dideoxy-adenosine (ddA). As shown in A, some SCs displayed high levels of cell surface O1 even in the presence of SQ22536. However, these cells did not convert into myelinating cells, as denoted by their multiple processes (indicative of failure to establish a one-to-one association with axons) and lack of MBP expression. The dose dependency of P-PKA substrate expression by increasing concentrations of SQ22536 was confirmed by western blot analysis in SC-only cultures (C). The arrowheads in B indicate a source of P-PKA substrate immunoreactivity exclusively present in differentiating O1 positive cells (boxes) that was insensitive to inhibition by SQ22536.

cAMP fulfills all basic requirements for a candidate intracellular signal that initiates SC differentiation in connection to myelin formation. To begin testing whether cAMP was required for myelination in ascorbate-treated SC-neuron cultures, we blocked the endogenous production of cAMP by addition of SQ22536, a widely used tmAC antagonist that reduces cAMP biosynthesis in response to a variety of GPCR ligands [23]. Results indicated that treatment with SQ22536 dose-dependently reduced the appearance of O1 positive and MBP positive cells (Fig. 7A-B). Provision of SQ22536 reduced O1 expression while preserving SC density (Fig. 7A, upper panels, DAPI channel) and neuronal/axonal integrity (Fig. 7A, neurofilament channel). Despite some cells were able to express O1 in the presence of SQ22536, these O1 positive cells consistently failed to elevate their expression of MBP and undergo the morphological changes typical of myelinating cells (Fig. 7A, middle and lower panels). The inhibition of O1 expression by 2’,5’-dideoxy-adenosine (ddA), another widely used inhibitor of the tmAC [31], served to confirm the antagonistic effect of SQ22536 (Fig. 7B).

To serve as a control for the action of SQ22536, we determined the dose dependency of P-PKA substrate inhibition by SQ22536 in isolated SCs by means of western blot analysis (Fig. 7C). The inhibition of P-PKA substrate expression by SQ22536 in axon-related SCs was confirmed by immunofluorescence microscopy in ascorbate-treated SC-neuron cultures (Fig. 7D). Strikingly, the strong cytoplasmic P-PKA substrate signal distinctive of O1 positive cells was insensitive to inhibition by SQ22536 (Fig. 7, right panel), which suggested the existence of a tmAC-independent source of cAMP in differentiating axon-associated SCs.

### The tmAC agonist forskolin mimicked the effect of cAMP analogs on O1 and MBP expression in axon-related SCs

**Fig 8:**
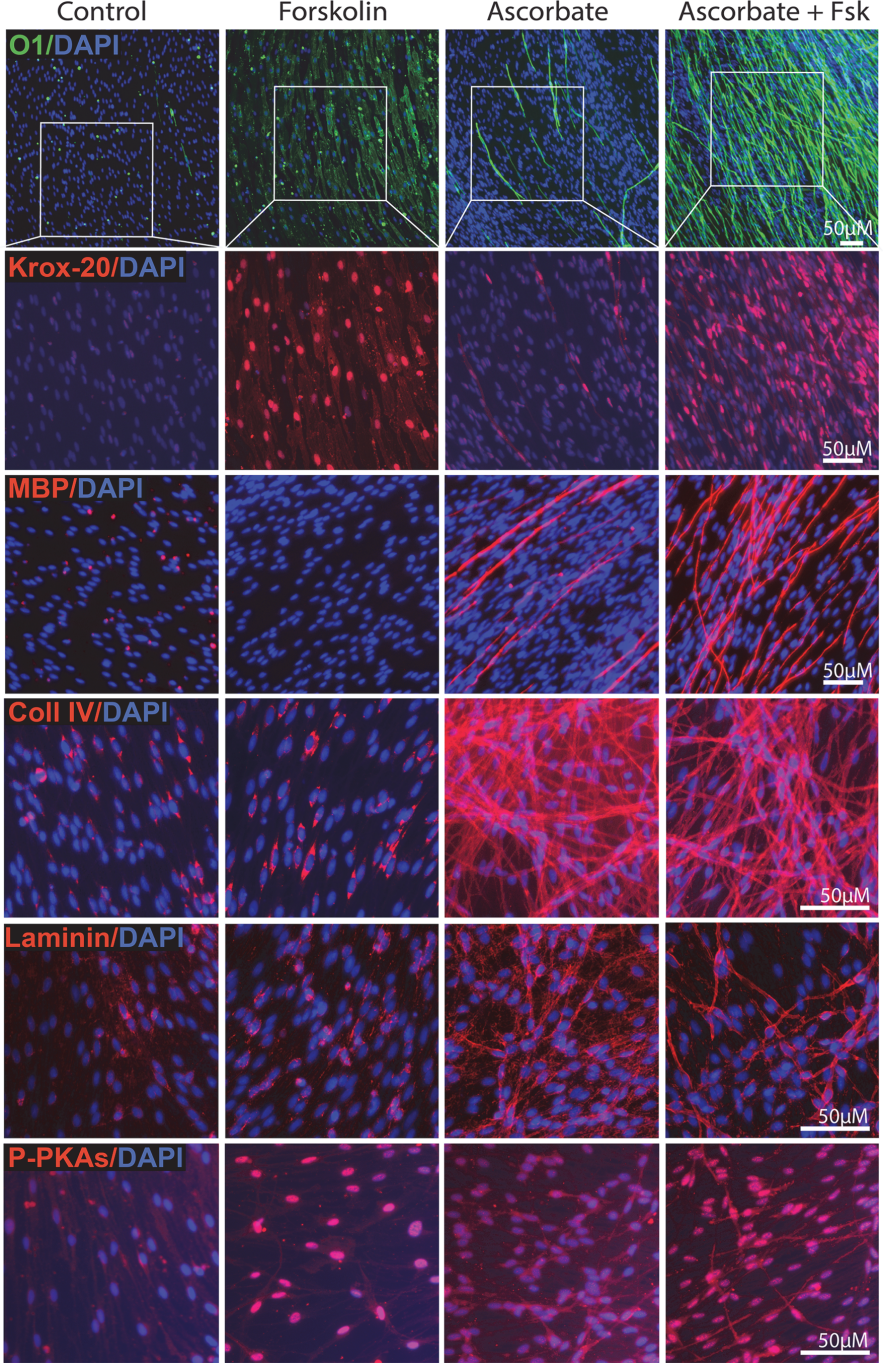
Expression of markers of differentiation and basal lamina in SC-neuron cultures treated with forskolin and ascorbate provided alone and in combination. The overall experimental design and analysis by immunofluorescence microscopy matched the one of Fig. 5 with the exception that forskolin (0.5 μM) was provided to the culture medium instead of CPT-cAMP. Cultures were stained with antibodies against O1, Krox-20, MPB, collagen type IV, laminin and P-PKA substrates, as indicated. High magnification images are shown for all markers. The areas demarked by the white boxes (upper panels) were enlarged in the panels below for better visualization of Krox-20 expression. Similar to CPT-cAMP, forskolin effectively enhanced the expression of Krox-20 and O1 but not that of MBP, collagen IV or laminin despite effectively increasing the phosphorylation of PKA substrates in all cells. In the presence of ascorbate, forskolin consistently enhanced MBP expression without further increasing the expression of basal lamina constituents.

**Fig 9:**
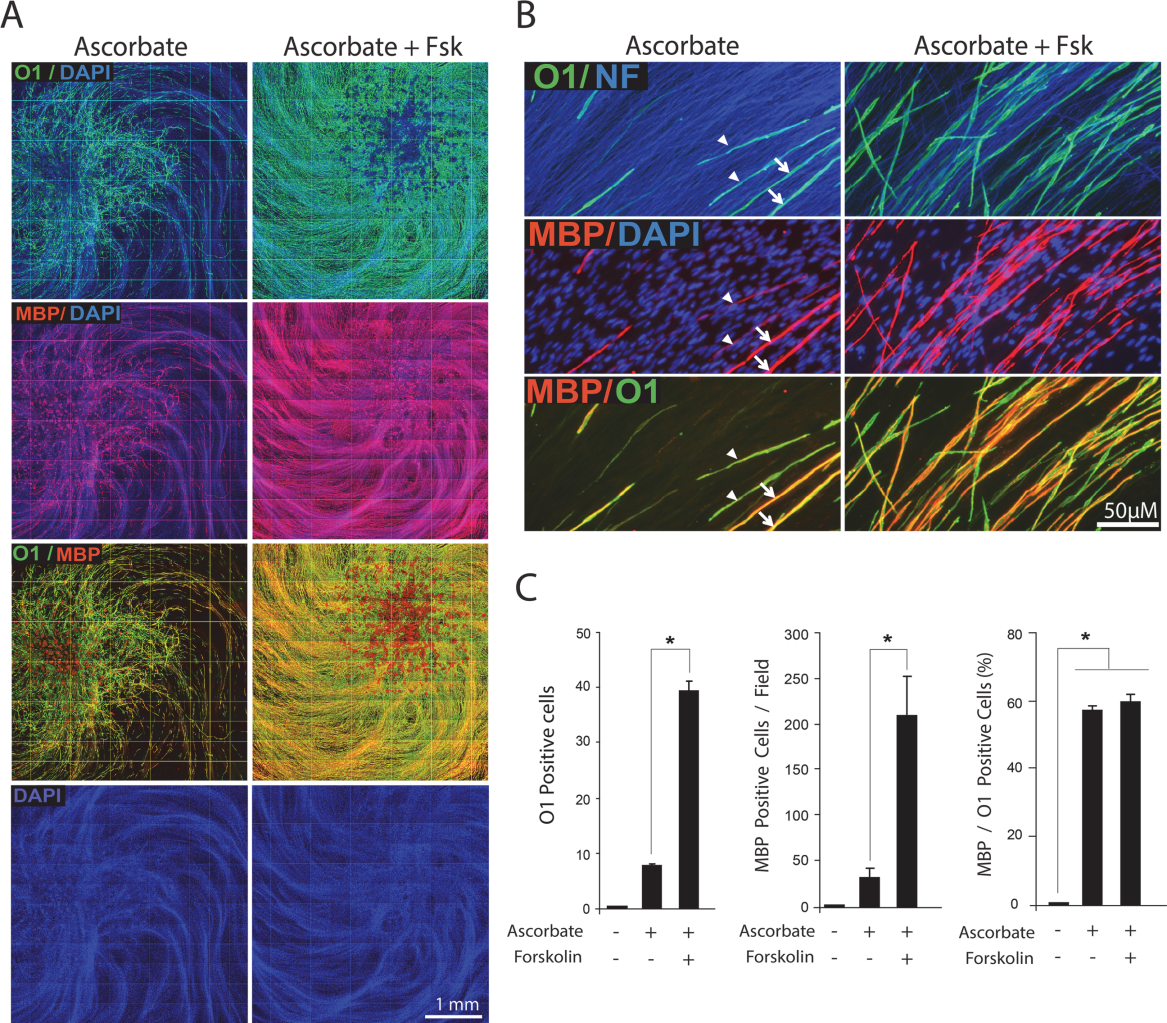
Induction of O1 and MBP expression by combined administration of forskolin and ascorbate: spatial distribution and quantitative analysis of O1 and MPB positive cells. Forskolin was provided to SC-neuron cultures together with ascorbate essentially as described in Fig. 8. Low magnification composites of representative cultures (A) are shown along with selected high magnification areas (B) to reveal the effect of the indicated treatments on O1 and MBP expression. The quantitative analysis provided in panel C confirmed that similar to CPT-cAMP, forskolin effectively enhanced O1 and MBP expression without concurrently increasing the MBP/O1 ratio. The DAPI channel in A (bottom panel) is shown to serve as an indication of unchanged cell density in forskolin-treated cultures. The arrowheads and arrows (B, left panels) point out to representative O1 positive cells exhibiting thin and thick myelin, respectively, as denoted by the intensity of MBP positive myelin segments. This heterogeneity of MBP staining in individual cells was not evident in forskokin-treated cultures (B, right panels), possibly due to increased synchronization in the myelination process.

Experiments using mtAC inhibitors provided pharmacological evidence indicating that the SC’s conversion into O1 positive cells was at least in part dependent on tmAC activity. Thus, we next used forskolin, a direct specific agonist of the tmAC, to examine the potential pro-differentiating effects of selective mtAC activation in SC-neuron cultures. Our previous studies in isolated SCs indicated that forskolin is a potent inducer of proliferation and PKA activity but a poor inducer of differentiation [11]. Whereas forskolin promotes nearly maximal expression of Krox-20, it fails to mimic the action of CPT-cAMP in driving the conversion into an O1 positive state independently of the concentration used [11]. In SC-neuron cultures, on the contrary, low doses of forskolin (0.5 μM) were sufficient to massively enhance not only Krox-20 but also O1 expression regardless of the presence of ascorbate (Fig. 8, upper panels). Whereas forskolin on its own failed to directly induce the expression of MBP and basal lamina constituents (Fig. 8, middle panels), it overly amplified the number and distribution of MBP positive cells when provided in combination with ascorbate (Figs. 8–9). Similar to CPT-cAMP, forskolin did not have an effect on collagen type IV or laminin synthesis (Fig. 8). In Fig. 8 (lower panels), the P-PKA substrate profile is provided for all conditions simply as a control for the effectiveness of forskolin treatment in SC-neuron cultures, as low concentrations of this drug (0.5 μM) were used for stimulation. A strong, mainly nuclear, P-PKA substrate labeling was observed in all forskolin-treated SCs, clearly indicative of widespread tmAC activation throughout the SC population.

A quantitative analysis of forskolin’s effects in ascorbate-treated SC-neuron cultures indicated that similar to CPT-cAMP, forskolin was sufficient to enhance O1 and MBP expression without changing the MBP/O1 ratio (Fig. 9C) or enhancing SC proliferation, as evidenced by the unchanged overall density of DAPI labeling throughout the culture system (Fig. 9A, lower panel) and EdU incorporation assays (not shown). TEM analysis further confirmed that similar to CPT-cAMP and contrary to ascorbate, forskolin did not increase the ensheathment of axons with cytoplasm or the formation of a basal lamina (not shown). The failure of forskolin to induce a basal lamina was consistent with the lack of effect of this drug on collagen type IV and laminin expression (Fig. 8).

### Forskolin increased both Krox-20 and O1 expression in axon-related SCs but only Krox-20 in axon-deprived SCs

**Fig 10:**
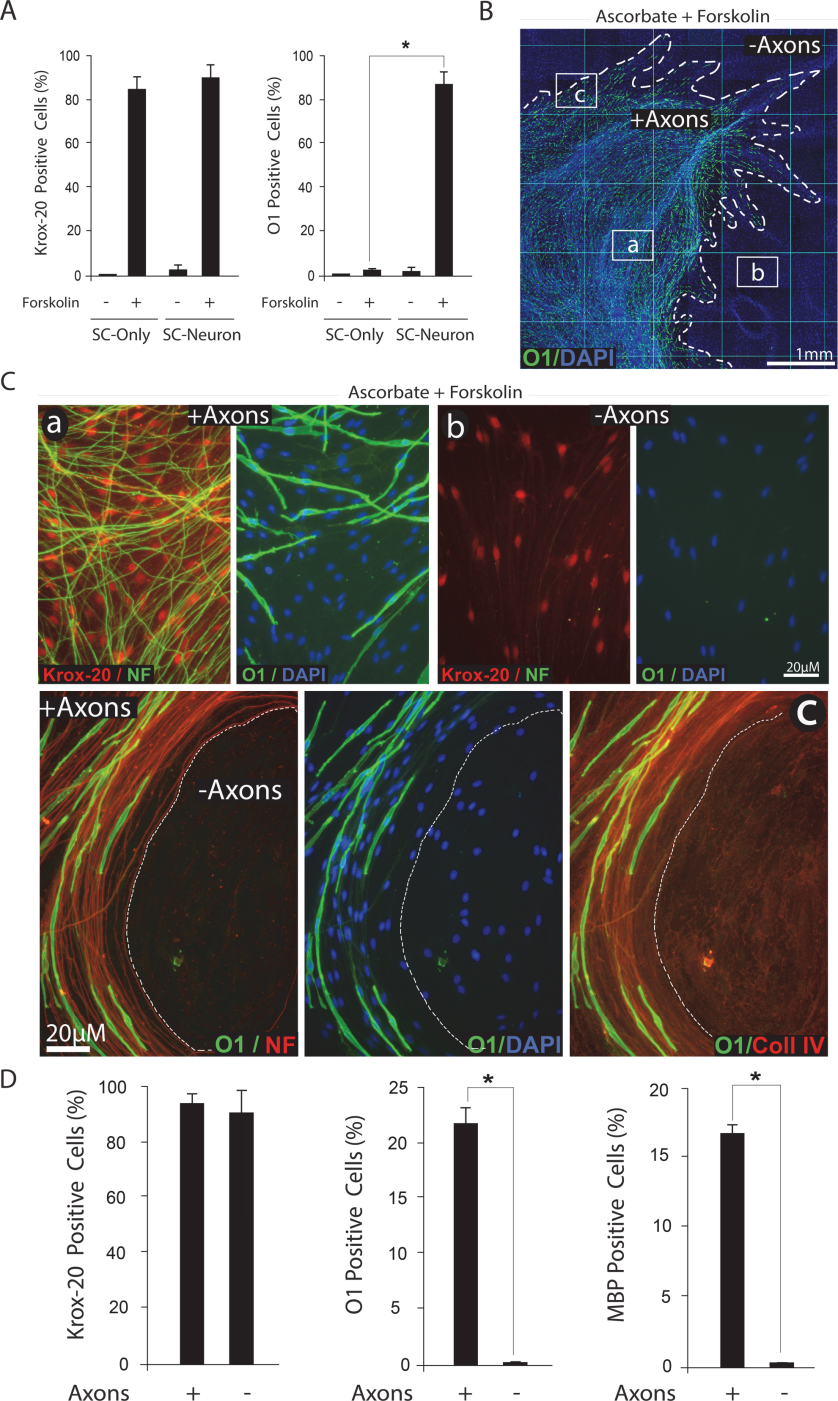
Induction of Krox-20, O1 and MBP expression by combined administration of forskolin and ascorbate: dependency on axonal contact. Experiments in SC-neuron cultures were identical to those described in Fig. 8, with the exception of experiments shown in panel A, where the responses of isolated (SC-only) and axon-related SCs (SC-neuron) were compared at 3 days post-stimulation. Representative areas located within (a), outside (b) and at the frontier (c) of the axonal outgrowth are shown at high magnification to reveal details of the morphology of the cells, their relationship to axons (neurofilament staining, NF) and their expression of Krox-20, O1 and collagen type IV, as indicated. In panel B, a low magnification image of a representative SC-neuron culture treated with ascorbate and forskolin is shown to provide a reference to the relative location of the areas displayed in C. Note the selective distribution of O1 positive cells (green) with respect to the total number of cells (DAPI) and the extension of the neurite outgrowth (dotted lines). A quantification of the percentage of Krox-20, O1 and MBP positive cells in selected areas within (+ axons) and outside (- axons) the axonal outgrowth is provided in panel D. Whereas Krox-20 expression was enhanced throughout the culture system, the expression of O1 and MBP was confined to those SCs that physically interacted with axons (C). Note that even those SCs that do not contact axons exhibit a profile of extracellular collagen IV expression (C, lower panels).

To understand the underlying basis for the apparently contradictory findings on the effect of forskolin in SC-only and SC-neuron cultures, we compared the expression of various differentiation markers in forskolin-treated SCs growing in an axon-containing and an axon-free environment, including the axon-deprived areas of SC-neuron cultures (Fig. 10). First, we confirmed that in isolated SCs, treatment with a low dose of forskolin induced Krox-20 expression without a concurrent increase in O1 (Fig. 10A). Second, we confirmed that similar to isolated SCs, Krox-20 expression occurred throughout the culture system, i.e. without regard to the location of the axons (Fig. 10C-D) and regardless of ascorbate administration (Fig. 8, upper panels). Third, we confirmed that the expression of O1, rather than Krox-20, was restricted to those SCs that established a direct physical contact with DRG axons (Fig. 10C-D). The observation that SCs deprived of direct contact with axons fail to undergo differentiation into an O1 state upon forskolin administration was consistent with similar observations in SC-only cultures [11]. Interestingly, SCs located outside the axonal web exhibited a pattern of extracellular collagen type IV expression that was roughly similar to the one exhibited by axon-contacting SCs (Fig. 10C). Because this pattern of collagen IV expression is consistent with the formation of a basal lamina [17] and still those SCs did not express O1, we conclude that axon contact rather than basal lamina formation is the limiting factor for effective O1 expression.

The comparison of the differentiating effect of CPT-cAMP, which generates a widespread increase in intracellular cAMP within the cells, with that of forskolin, which limits the production of cAMP to the activation of the endogenous tmAC, led to the following interpretation of results: (1) The activation of the tmAC was sufficient to prime axon-related SCs but not axon-deprived SCs to differentiate into O1 positive cells, most likely via directly increasing the expression of Krox-20; (2) The activation of the tmAC was not sufficient to directly induce O1 expression, basal lamina formation or myelination; and (3) Additional molecular signals emanating from axons rather than the basal lamina, possibly acting via cAMP, were required for full SC differentiation into an O1 state.

### sAC, a novel AC and bicarbonate sensor, provides an unconventional source of cAMP essential for SC differentiation

Traditionally, the only known source of cAMP was the family of tmAC isoforms which are responsive to the activation of GPCRs and heterotrimeric G proteins as well as the non-physiological activator forskolin [24]. The more recent discovery of sAC, a highly conserved and ubiquitous form of AC localized in the cytoplasm, organelles, and nucleus of eukaryotic cells, has indicated the existence of an alternative source of cAMP biosynthesis that is not limited to the plasma membrane [25]. Contrary to the tmAC, sAC is insensitive to forskolin and GPCR agonists and its activity within cells is directly modulated by changes in the intracellular levels of bicarbonate, calcium and ATP [26]. Similar to the tmAC, sAC activation enhances PKA activity and gene expression [27], albeit through a poorly defined mechanism of action. Because sAC is insensitive to activation by forskolin and GPCR ligands, we reasoned that this novel AC could be a likely candidate to mediate the action of cAMP in driving the SC’s transition into an O1 state. Thus, we performed a series of immunostaining and western blot experiments aimed at detecting sAC expression and activity in SCs. Experiments were carried out under basal conditions (control) as well as conditions in which sAC activity was stimulated by treatment with its physiological activator bicarbonate or inhibited by treatment with pharmacological sAC antagonists.

**Fig 11:**
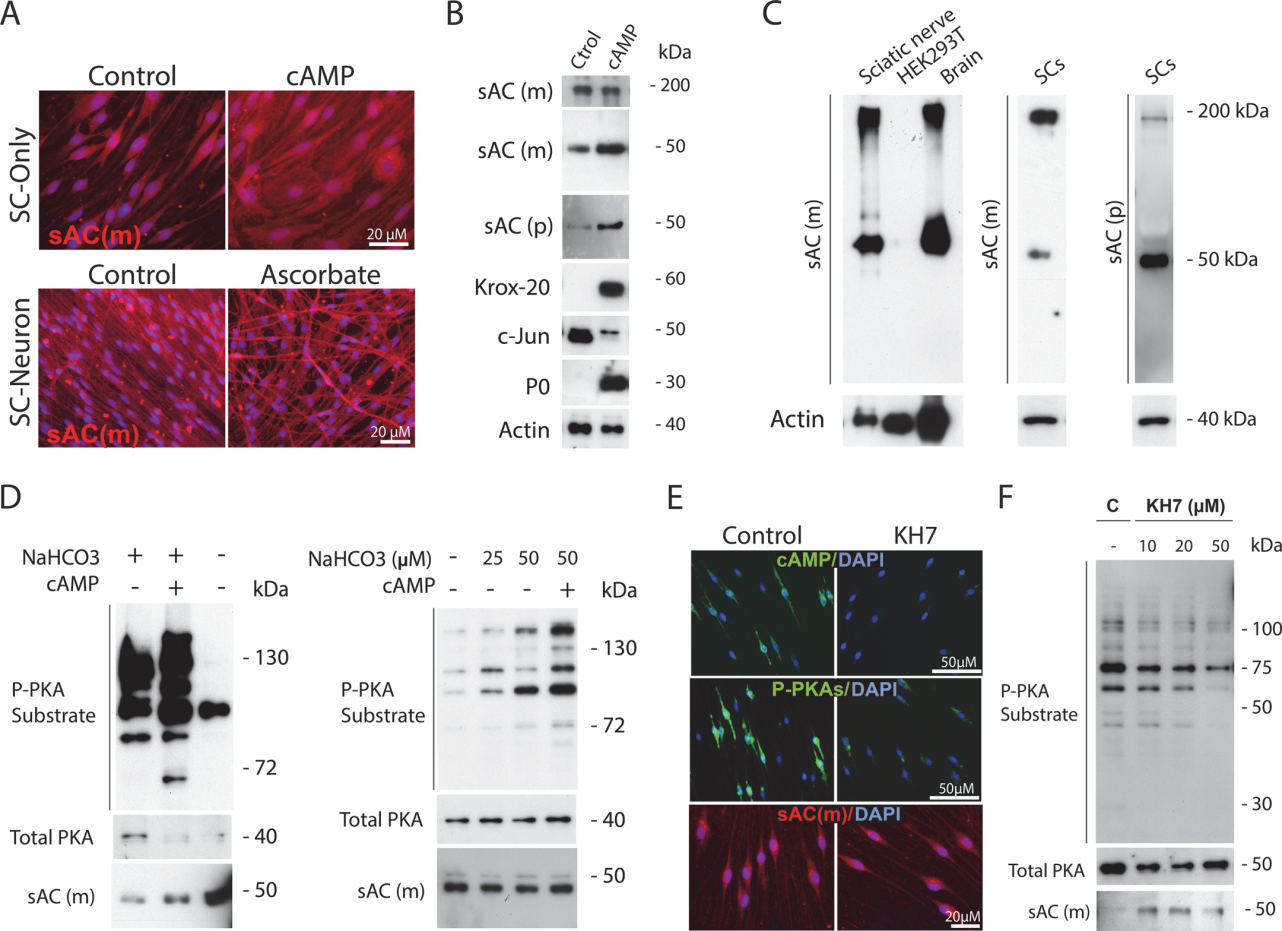
Expression and activity of sAC in cultured SCs: changes with differentiation, responsiveness to bicarbonate stimulation and pharmacological inhibition with KH7. In panel A, sAC expression was detected in non-differentiated (control) and differentiated SCs (i.e. cells treated with CPT-cAMP, 250 μM, for 3 days) and SC-neuron cultures non-treated (control) and treated with ascorbate for 12 days. Western blot analysis of control and cAMP-treated SCs with two different anti-sAC antibodies (a sAC monoclonal, sAC-m, and a sAC polycolnal, sAC-p) revealed the expression of two distinct sAC immunoreactive bands that migrated at ~180 kDa (full length sAC) and ~50 kDa (truncated sAC isoform), respectively. The ~50 kDa protein was increased upon treatment with cAMP (B). In panel C, the specificity of sAC detection was confirmed in samples of purified SCs, sciatic nerve extracts, brain extract (positive control) and HEK293T cells (negative control). In D (left panel), SCs growing in bicarbonate-containing DMEM medium (alone or together with CPT-cAMP, 250 μM) or bicarbonate-free DMEM medium for 3 days were collected for western blot analysis using antibodies against P-PKA substrates, total PKA and sAC. In D (right panels), SCs were incubated in bicarbonate-free DMEM for 24 hours and then, stimulated with the indicated concentrations of sodium bicarbonate (NaHCO3) alone or together with CPT-cAMP for an additional 24 hour period. In panels E-F, SC-only cultures were starved of mitogens and serum for 3 days and left untreated (control) or treated with KH7, which was provided at 20 μM unless otherwise indicated. Cells were treated for 30 min (E) and 24 hours (F) prior to analysis by immunofluorescence microscopy and western blot, respectively. In E, cultures were analyzed for their immunoreactivity with antibodies against cAMP (upper panels), P-PKA substrates (middle panels) and sAC (lower panel). The high levels of cAMP and P-PKA substrate expression in non-stimulated SCs, along with their sensitivity to bicarbonate stimulation and KH7 inhibition, are indicative of constitutive sAC activity.

**Fig 12:**
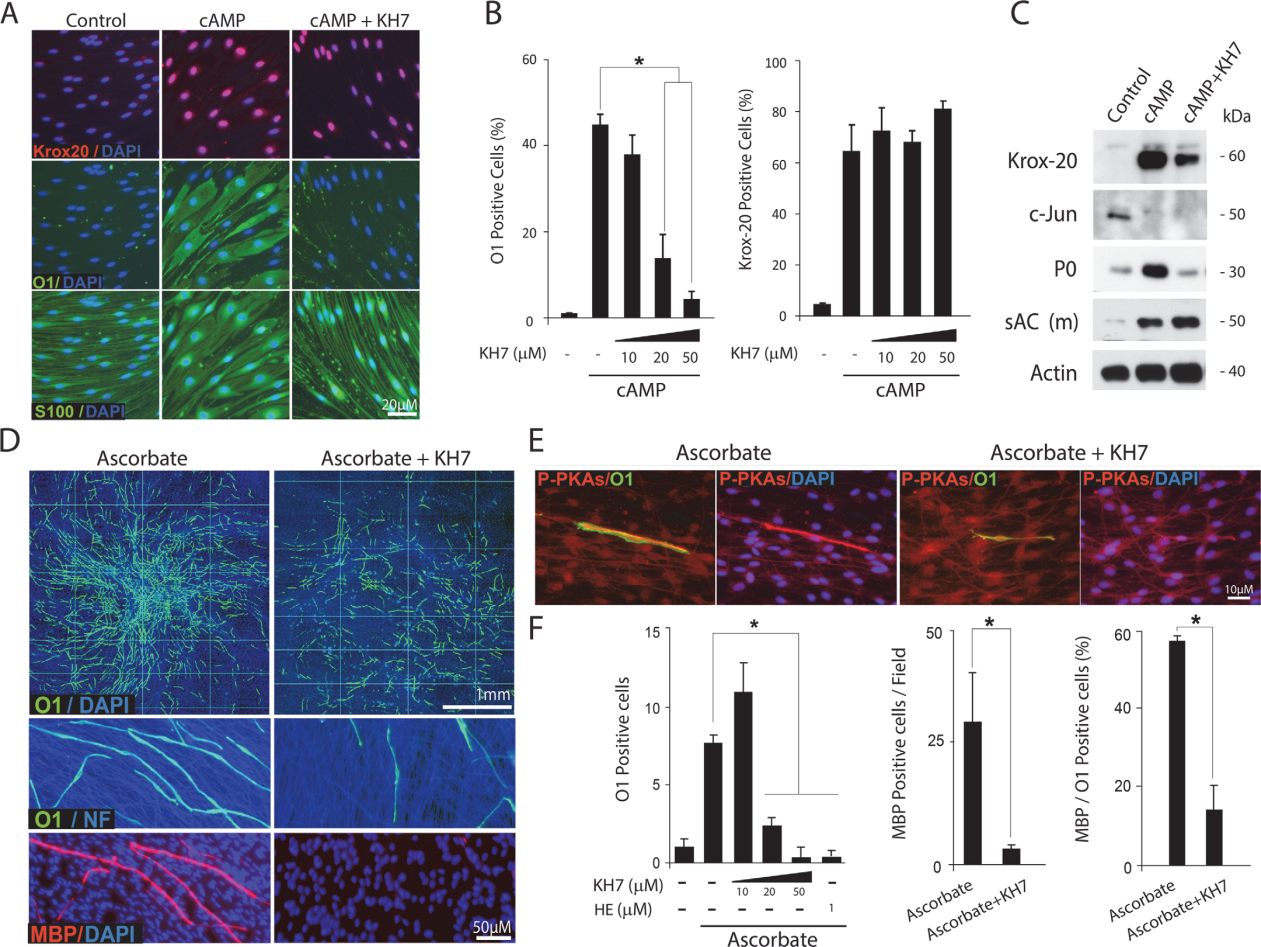
Inhibition of O1 expression and morphological differentiation by pharmacological sAC antagonists. The experimental design and analysis of results carried out using SC-only (A-C) and SC-neuron cultures (D-F) were identical to those of previous figures. In panels A-C, isolated SCs were left untreated (control) or treated with cAMP (CPT-cAMP, 250 μM) in the absence or presence of increasing doses of KH7, as indicated. In panels D-F, SC-neuron cultures were stimulated with ascorbate-containing medium in the absence (control) or presence of KH7, which was used at 20 μM unless otherwise stated in the figure. Cultures were double-immunostained with antibodies against O1 and MBP (D) or O1 and P-PKA substrates (E). Data presentation and quantitative analysis of Krox-20, O1 and MBP expression was done as previously described. In panel F, a condition that used 2-hydroxy-estradiol (HE) was included in the quantitative analysis of O1 expression for confirmation of results. KH7 abrogated the morphological transformation associated with the differentiation of isolated (A, S100 expression) and axon-related SCs (D-E, O1 expression). KH7 diminished the total levels of expression of O1 (A-B and D-F), P_0_ (C) and MBP (D-F) without reducing the expression of Krox-20 (A-C) or increasing that of c-Jun (C).

Experiments shown in Fig. 11 collectively indicated that cultured SCs predominantly expressed the full-length sAC (~180 kDa) and a truncated sAC isoform (~50 kDa) whose expression has also been described in a variety of cell types, including cultured neurons [28]. Results were confirmed by means of two different sAC antibodies, an anti-sAC monoclonal (R21) that recognizes the N-terminus of sAC [29] and an anti-sAC polyclonal antibody from a commercially available source. sAC expression was detected in cultures of non-differentiated and cAMP-differentiated SCs (Fig. 11A-B), SC-neuron cultures treated and non-treated with ascorbate (Fig. 11A) and adult sciatic nerve tissue (Fig. 11C). The full western blot profiles of immunoreaction with the sAC antibodies mentioned above showed no other evident immunoreactive bands besides the ~180 and ~50 kDa proteins under standard denaturing conditions of electrophoresis. These two bands co-migrated with those from brain extract, which was used as positive control for sAC expression (Fig. 11C). In addition, no signal was detected in a HEK293T cell lysate, which was used as negative control, altogether lending confidence on the specificity of the antibodies used. Immunofluorescence staining of fixed cells revealed that the immunoreactive sAC signal predominantly localized to the cytoplasm (perinuclear region) and processes of cultured SCs regardless of their state of differentiation (Fig. 11A). Interestingly, prolonged cAMP treatment increased sAC expression (predominantly the ~50 kDa isoform) along with the expression of myelination markers such as Krox-20 and P_0_ (Figs. 11A-B and 12C).

Functional evidence of constitutive sAC activity in SCs was suggested by starvation experiments which indicated that SCs synthesize cAMP, and thereby maintain high basal levels of PKA activity, even in the absence of exogenously provided GPCR/mtAC ligands. The combined results from immunofluorescence microcopy and western blot analysis presented in Fig. 11 showed that SCs exhibited relatively high levels of cytoplasmic cAMP (Fig. 11E) and PKA activity (Fig. 11D-F) even after prolonged removal of mitogens and serum. Additional evidence for sAC activity in SCs was provided by experiments which tested the effect of bicarbonate addition and removal on P-PKA substrate expression [30]. These results, which are reported in Fig. 11D, confirmed that the removal of bicarbonate from the culture medium reduced whereas the addition of bicarbonate dose dependently increased the P-PKA substrate signal in mitogen-starved SCs. Furthermore, the basal levels of intracellular cAMP and P-PKA substrate immunoreactivity in non-stimulated SCs were sensitive to inhibition by KH7 (Fig. 11E-F), a pharmacological sAC antagonist deemed specific to discriminate between tmAC and sAC activities [31]. The activity of sAC rather than the levels of sAC expression (~50 kDa isoform) was dose dependently reduced in the presence of KH7 (Figs. 11E-F and 12C). Of note, KH7 was used in a concentration range that is known to selectively antagonize the activity of a neuronal ~50 kDa isoform of sAC [28].

Based on the immunological and pharmacological evidence collectively suggesting constitutive sAC bioactivity in SCs, we next chose to use KH7 to test the hypothesis that sAC-derived cAMP was required for SC differentiation. Immunostaining and western blot analysis confirmed that KH7 dose dependently prevented the induction of O1 (Fig. 12A-B) and P_0_ expression (Fig. 12C) while preserving relatively high levels of nuclear Krox-20 (Fig. 12A-B) and low levels of c-Jun (Fig. 12C). In SC-neuron cultures, KH7 dose dependently reduced both O1 and MBP expression (Fig. 12D and F) without affecting the health of the SCs or the DRG axons, as judged by the total cellular density of the co-cultures (Fig. 12D, DAPI channel) and staining with neurofilament antibodies (Fig. 12D), respectively. The antagonistic effect of KH7 on O1 expression was confirmed by using the catechol estrogen 2-hydroxy-estradiol (HE), which is also deemed to be a selective antagonist of sAC [32], (Fig. 12F). Strikingly, KH7 not only prevented O1 expression but also effectively antagonized the transformation in cell size and shape that SCs undergo upon differentiation with cAMP (Fig. 12A) and myelination with ascorbate (Fig. 12D). Of note, KH7 abrogated the cytoplasmic P-PKA substrate labeling typically found in axon-associated differentiating SCs (Fig. 12E), indicating the dependency on sAC rather than mtAC activity.

Altogether, these results suggest a contribution of sAC activity to the intracellular levels of cAMP in SCs and a requirement for differentiation. We understand that the concerted and probably sequential activation of tmAC and sAC contribute to the onset of myelination essentially by driving the SC’s transition into an O1 positive stage. This transition seems to be the most relevant cAMP-dependent rate limiting step that restricts the SC’s conversion into myelin-forming cells.

## Discussion

### A mechanistic model for the early control of SC differentiation by AC-cAMP signals

The results presented herein are consistent with the idea that the AC/cAMP signaling system plays a key role in differentiation prior to and possibly independently of the onset of myelination. As such, we found that cAMP did not replace the action of ascorbate towards initiating axon ensheathment, basal lamina formation and myelin membrane wrapping. Still, cAMP strongly primed SCs to form myelin most likely through its already described activity to drive cell cycle exit, Krox-20 expression and initial differentiation into an O1 positive state. The maturation of axon-related SCs into a more advanced state of differentiation denoted by the induction of MBP expression and the formation of a myelin sheath per se, required an axonal environment and ascorbate supplementation, whose combined effects could not be mimicked in whole or in part by exogenously added cAMP-stimulating agents such as CPT-cAMP or forskolin.

**Fig 13:**
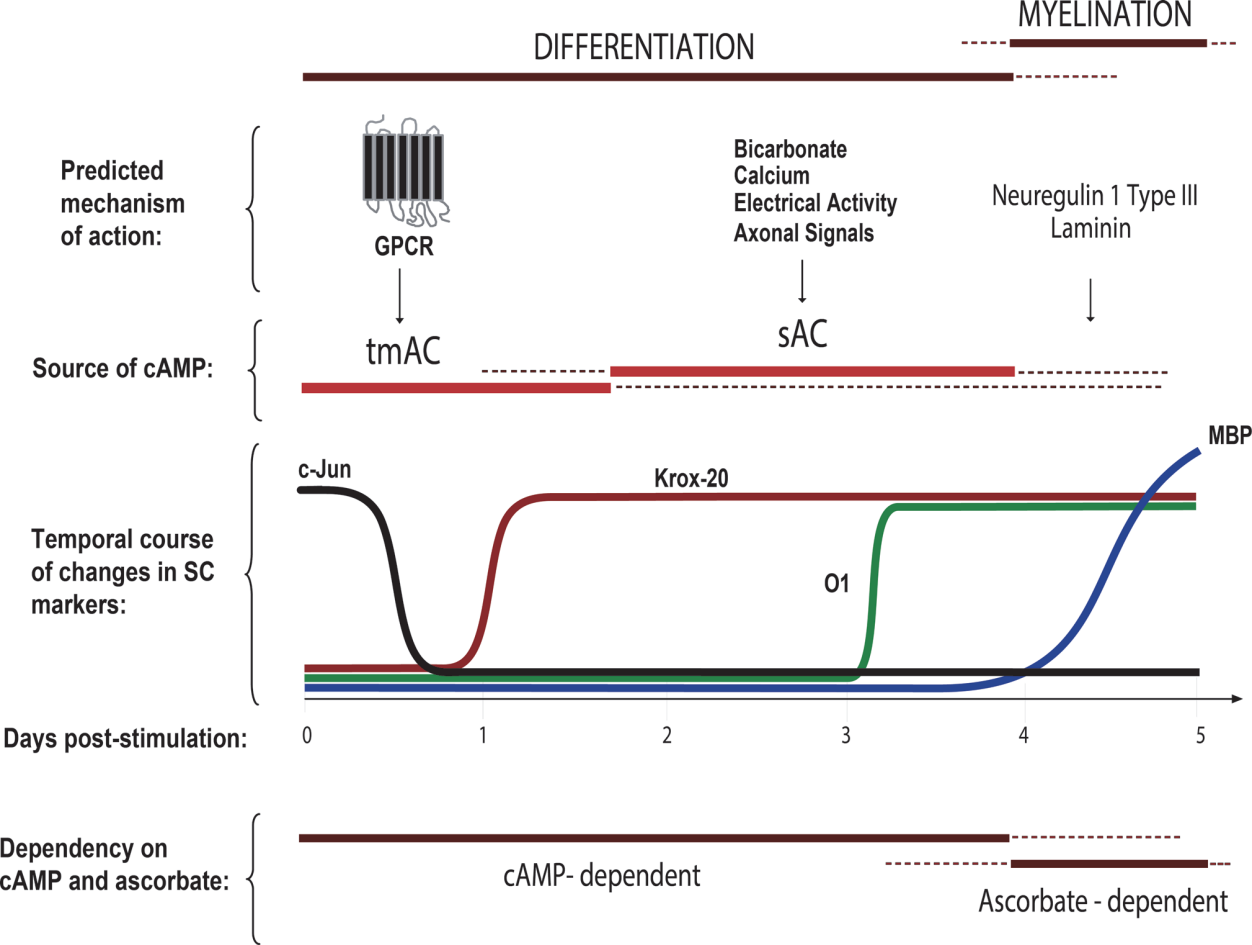
Requirement of cAMP signaling restricts the onset of myelination. Our cell culture studies revealed a crucial role for GPCR-sensitive and GPCR-insensitive sources of cAMP (namely tmAC and sAC) in driving SC differentiation into an O1 phenotype, a stage that precedes MBP expression and the formation of myelin sheaths. For practical purposes, we selected the markers Krox-20, O1 and MBP as phenotypic indicators of the early, mid-term and later stages of differentiation, respectively. We propose a mechanistic model that predicts an orderly requirement of signals from activated Gαs-coupled GPCRs (e.g. Gpr126), sAC (e.g. electrical activity, bicarbonate, and axonal signals), axon contact (e.g. neuregulin type III) and/or basal lamina components (e.g. laminin) prior to the initiation of myelin membrane wrapping. Data also suggests that sAC activation in SCs may depend on yet-to-be-identified axonal/neuronal factors.

One important aspect of cAMP’s mode of action in isolated and axon-related SCs was that it served to effectively synchronize the cellular responses to a differentiating stimulus. This synchronization allowed us to observe that the changes in the expression of Krox-20, O1 and MBP occured in an orderly temporal sequence, insofar as the induction of Krox-20 occured within 1 day, that of O1 within 3–4 days and that of MBP within at least 5–7 days after stimulation with the appropriate inductive signals. The sequence of this progression was reflected by the simple observation that not all Krox-20 positive cells labeled positive for O1 and not all O1 positive cells labeled positive for MBP, at least during the initial stages of differentiation in cell culture. Thus, the appearance of the Krox-20, O1 and MBP positive phenotypes, respectively, seemed to represent different stages of differentiation of further complexity towards the myelinating phenotype. Most importantly, our studies have indicated that the abovementioned phenotypic transitions seem to rely on distinct regulatory mechanisms. The novel findings and conclusions derived from these series of studies using cultured cells are illustrated in the schematic diagram shown in Fig. 13.

### Cooperation between classical and novel ACs in the early control of SC differentiation

Experiments using a combination of type-selective pharmacological AC agonists and antagonists provided evidence indicating that the onset and progression of SC differentiation relies on cAMP signals derived from at least two different sources: 1) The tmAC, which seems to be sufficient to increase Krox-20 and down-regulate c-Jun expression; and 2) The sAC, which is in turn required for O1 expression and morphological differentiation of the cells.

The requirement of tmAC and sAC activities for driving the O1 phenotype is indicative of a complex molecular mechanism of regulation by extra- and intra-cellular signals. The mechanistic model presented in Fig. 13 predicts an initial wave of cAMP induction through a GPCR/Gαs/tmAC-dependent signal along with a second or concomitant wave of cAMP biosynthesis initiated by sAC. We may speculate that activated Gαs-coupled GPCRs such as the newly discovered Gpr126 may drive Krox-20 expression and initiate differentiation through activation of the tmAC. In support of this concept, Gpr126 was recently shown to induce cAMP via a Gαs-dependent mechanism of action [14]. The finding that forskolin restores Krox-20 expression and rescues the myelination defect in Gpr126 knock-out zebrafish is also in agreement with this idea [15]. Consistent with our findings, it was also shown that transient cAMP elevation with forskolin is sufficient for Krox-20 expression and myelination in the absence of Gpr126 signaling [12], which implies that the maintenance of Krox-20 expression and myelin maturation both occur independently of Gpr126 signaling and consequently, independently of cAMP.

Multiple lines of evidence suggest that mtAC activation is not a sufficiently potent signal to drive myelination. Of all the GPCR agonists that we have shown active at eliciting cAMP changes in SCs, including epinephrine and adenosine [33], none have displayed activity on their own to induce O1 expression (not shown). Even though results using forskolin suggest that mtAC activity plays a role at the very onset of differentiation, whether tmAC activity is dispensable for later stages of differentiation is uncertain. Our experiments in cultured cells have revealed that the activation of the tmAC alone may be sufficient to increase the ratio of Krox-20 to c-Jun expression but not to promote the O1 positive phenotype in the absence of other signals, as evidenced by the inability of forskolin to display a full differentiating activity in isolated SCs and SCs deprived of axon contact. Still, selective tmAC activation with forskolin is an apparently sufficient pro-myelinating signal in those SCs that are directly exposed to contact with axons, which suggests the existence of putative axonal signals that cooperate with tmAC to drive SC differentiation. Our results indicate that such axonal signals may work at least in part though activating sAC in SCs. The finding that SCs express sAC, along with its requirement for differentiation, not only emphasizes the relevance of cAMP but also suggests that myelination may be controlled by an unconventional mechanism of action that is independent of GPCR-Gαs signaling. One candidate axon-derived sAC-activating signal known to directly increase myelin formation is electrical activity, as action potentials are essential to promote local translation of MBP mRNA in central myelinating glia [34].

Even though sAC is likely to be involved in a variety of cellular functions, its role in somatic cell differentiation has only been reported in a few different cell types, including osteoclasts [35], PC12 cells [36] and retinal ganglion neurons [28]. Albeit the use of pharmacological inhibitors may raise some concerns due to possible off-target effects, we understand that we have used a standard pharmacological and immunological approach to tackle the potential contribution of sAC in our cellular system [31]. Still, more in depth signal transduction studies to address the differential role of mtAC vs sAC on myelination, including gene down-regulation studies and confirmation in an *in vivo* system of peripheral myelination, are necessary to further test the consistency of our mechanistic model and the physiological significance of our data. Nevertheless, our combined results derived from the use of so deemed type-selective agonists and antagonists of mtAC and sAC activities lend confidence on our conclusion that an unconventional source of cAMP, namely sAC, is required for full differentiation of cultured SCs.

### Contribution of cAMP to axon contact- and basal lamina- induced myelination

A long-standing concept in the field of glial cell development is that myelination occurs in response to axon contact-dependent signals that allow the formation of a basal lamina, a structure without which myelination does not occur [37]. Cell-to-cell interactions between SCs and axons are essential for the ensheathment of axons and the proper assembly of the myelin sheath [21,38, 39]. Surprisingly, our data indicates that SCs can acquire a differentiated, pre-myelinating phenotype through a process that is independent of the one that controls myelination. Extensive data has shown that the levels of axon-bound neuregulin-1 type III are key determinants for regulating myelin thickness in peripheral nerves [2, 40]. In spite of the increasing complexity underlying neuregulin/ErbB signaling networks, it is unlikely that neuregulin drives myelination via cAMP, as tyrosine kinase receptors in general do not typically transduce signals through the cAMP second messenger system. In fact, evidence suggests that neuregulin/ErbB signaling may act at a later stage and likely independently of cAMP to control the myelination process. In support of this idea, a recent study of Gpr126 mutants has shown that overexpression of axonal neuregulin is not sufficient to initiate myelination in the absence Grp126 [12]. In turn, myelination does not proceed in the absence of ErbB signaling despite enhanced cAMP [12], which altogether suggests that cooperation instead of overlap between cAMP and neuregulin-ErbB signaling is likely to be required for myelination.

Another open question is whether neuregulin/ErbB signaling directly drives the expression of myelination-associated genes and/or integrates signals from other pathways. We have previously shown that neuregulin, which is also the most potent SC mitogen, does not mimic the effect of cAMP on shifting the Krox-20/c-Jun balance, inducing a growth arrested state or driving the expression of O1 in cultured SCs [8, 20]. It has been shown that neuregulin drives myelin sheath formation by increasing membrane spreading and motility around the axons through an ErbB-dependent mechanism that targets key proteins regulating actin dynamics [41]. These cytoskeletal changes along with the neuregulin-dependent regulation of cholesterol biosynthesis [42] may explain at least in part the positive effect of neuregulin on myelin thickness and its requirement for myelination.

### Uncoupling between differentiation and myelination in SCs

Standard myelinating conditions for SC-neuron cultures which use ascorbate supplementation as differentiating stimulus allow SCs to differentiate in an asynchronous manner. Even though ascorbate increases nuclear Krox-20 in an important fraction of the SC population, most of these cells fail to acquire O1 expression. Ascorbate dramatically increases axon ensheathment and facilitates basal lamina assembly by most SCs regardless of their relative location within the axonal web. Co-staining experiments revealed that only a small subset of collagen IV positive cells also express O1, indicating that basal lamina formation is not sufficient for SC differentiation into an O1 positive state. In addition, TEM experiments revealed that only a minor proportion of the ensheathing SCs initiate the wrapping of myelin membranes [17].

What are the factors limiting the onset of myelination? It has been argued that the signals controlling differentiation of central myelinating glia may differ from those controlling the process of myelination. In spite of their strict interdependence, differentiation and myelination may be functionally uncoupled and differentially controlled [43]. Our data suggests that a similar differentiation/myelination uncoupling may operate in SCs, as differentiation can be halted at different stages prior to the onset of the expression of MBP. Our studies provided evidence indicating that SCs encounter at least three discrete regulatory restriction points that limit the onset of myelination. An early restriction point in differentiation seems to be the cAMP-dependent transition into a Krox-20 positive stage, which may be dependent on tmAC activity. Yet, differentiation may not simply be a default cellular event that results from enhanced Krox-20 expression, as SCs can express maximal levels of nuclear Krox-20 without becoming O1 positive or morphologically differentiated [11]. The second restriction point seems to be the cAMP-dependent Krox-20-to-O1 transition, which seems to be, at least in part, dependent on sAC activity. The existence of this restriction point serves to explain why cAMP is such a powerful signal to induce O1 expression and overcome the limited myelinating capacity of *in vitro* cultured SCs. A third restriction point seems to operate at the O1-to-MBP transition, a process that may not directly depend on cAMP but on signals derived from the axons and/or the basal lamina, possibly neuregulin 1 type III and laminin (Fig. 13).

To conclude, our improved understanding of the mechanism of action of sAC and its coupling to tmAC signaling will provide important clues on the early steps limiting the SC’s progression into a myelinating state. A better understanding of how cAMP signals are integrated with other signals emanating from both the axonal and extracellular matrix environments will shed light on the early steps required for myelination as it occurs during development and repair *in vivo*.

## Materials and Methods

### Ethics statement

All procedures using animals were approved by the University of Miami Animal Care and Use Committee.

### Materials

CPT-cAMP [8-(4-Chlorophenylthio) adenosine-3',5'-cyclic monophosphate] was from Biolog (US distributor, Axxora LLC, San Diego, CA). Recombinant heregulin-β1_177–244_ (herein referred to as neuregulin) was from Genentech (South San Francisco, CA). Forskolin, KH7, anti-laminin and anti-actin antibodies were from Sigma (St. Louis, MO). MBP, P_0_ and neurofilament antibodies were from Chemicon (Temecula, CA). Antibodies against c-Jun and sAC (anti-ADCY10 goat polyclonal) were from Santa Cruz. Antibodies against collagen type IV were from AbDSerotec. SQ22536, KT5720, 2’,5’dideoxy-adenosine and 2-hydroxy-estradiol were from Calbiochem-Novabiochem Corp. (La Jolla, CA). Bicarbonate-free DMEM was from Life Technologies. The O1 hybridoma cells were kindly provided by Dr. M. Schachner (Rutgers, Piscataway, NJ), the Krox-20 polyclonal antibody by Dr. Dies Meijer (Erasmus University Medical Center, Rotterdam) and the sAC monoclonal antibody (R21) by Drs. Levin and Buck (Cornell University).

### Primary cultures of rat SCs and DRG neurons

SCs were obtained from the sciatic nerves of adult (3 month-old) female Fisher rats by a modification of a previously reported method [19]. Briefly, the sciatic nerve tissue was cut into small segments and allowed to degenerate *in vitro* by incubation for 10 days in DMEM medium containing 10% heat inactivated FBS (DMEM-10% FBS). Degenerated nerve explants were dissociated with a mixture of 0.25% dispase and 0.05% collagenase and the resulting cell suspension was plated on poly-L-lysine (PLL)-coated dishes in DMEM-10% FBS. Contaminating fibroblasts were removed by a complement reaction using Thy 1.1 antibodies (ATCC, Manassas, VA). The purified SCs were expanded up to passage one in DMEM-10% FBS medium supplemented with 2 μM forskolin, 20 μg/ml bovine pituitary extract (Biomedical Tech., Stoughton, MA) and 10 nM neuregulin. We have previously shown that SCs obtained by this method are fully competent to myelinate axons from dissociated DRG neurons in cell culture [19]. Experimental conditions were tested using early passage SCs (2 to 4 rounds of expansion) plated on PLL-laminin coated 24-well dishes. SC cultures consisted of >98% SCs based on double immunostaining with the SC-specific markers S100 and p75^NGFR^. For practical purposes (i.e. cell yields / preparation), experiments were performed using cultures of adult SCs. We have previously compared adult and postnatal rat SCs obtained by the method of Brockes [44] and found no relevant differences in their responses to cAMP or their capacity to form myelin *in vitro* [8, 20].

Cultures of purified embryonic dissociated DRG neurons were established following standard methods [45]. The DRG bodies were dissected from rat embryos on the 15^th^ day of gestation and then dissociated with 0.25% trypsin (37°C, 45 min) followed by gentle trituration. The resulting cell suspensions (50 μl drops containing 5,000–20,000 cells) were plated in the center of a well from a laminin-coated 24-well dish (BD Biosciences). Cultures were purified of non-neuronal cells by treatment with the anti-mitotic agent 5-fluoro-2'deoxyuridine (10 μM), which was provided in the cell culture medium one day after cell plating for 3 days (one cycle). The neuronal cultures were established and maintained in Neurobasal medium containing B27 supplement (Invitrogen, Carlsbad, CA), 25 ng/ml nerve growth factor (R&D Systems) and 1 mM L-glutamine (Life Technologies). DRG neurons were used for experimentation 10–15 days after plating. This method renders pure neuronal cultures with the neuronal somas located roughly in the center of the well and the axons radiating outwardly towards the periphery of the well.

### Differentiation assays using SC-only and SC-neuron cultures

SCs were induced to differentiate in response to treatment with phosphodiesterase-resistant cell permeable analogs of cAMP essentially as described in our previous publications [8, 11]. Throughout these studies, we provided the derivative CPT-cAMP to the culture medium at a concentration of 250 μM (isolated SCs) or 20 μM (SC-neuron cultures). The use of CPT-cAMP was preferred over that of other available cAMP analogs such as db-cAMP because of its negligible cytotoxicity, chemical stability and demonstrated potency to induce SC differentiation. SC cultures maintained from the onset in the absence of cAMP-inducing agents served as a control for undifferentiated (immature) cells [8]. Cultured SCs typically display an immature proliferative phenotype denoted by negligible levels of expression of myelin-associated proteins and lipids unless subjected to prolonged and persistent stimulation with cell permeable cAMP analogs. Briefly, purified SCs were plated and incubated in DMEM medium containing 10% FBS (no neuregulin or forskolin) for 1 day. The following day, the medium was changed to DMEM-1% FBS to deprive the cells of serum (1 day) prior to addition of vehicle (DMEM in control) or CPT-cAMP (prepared in DMEM) for 3 additional days, unless otherwise indicated. SCs in SC-neuron cultures were allowed to proliferate extensively for at least 7–10 days in response to axon contact stimulation (in the absence of cAMP-stimulating agents) prior to receiving treatment with CPT-cAMP or forskolin for the period indicated in the figure legends. In SC-neuron cultures, concentrations of CPT-cAMP 10 times lower than those required to induce differentiation in isolated cells were sufficient to promote a maximal response on O1 expression. Importantly, differentiation assays using SC-only and SC-neuron cultures were typically carried out simultaneously in parallel cultures so as to simplify experimental manipulation of the cells and interpretation of results. Cells were analyzed for the expression of differentiation markers by immunofluorescence microscopy or western blot as described below.

### Myelin formation assays using co-cultures of SCs and DRG neurons

Myelinating co-cultures of SCs and DRG neurons were established essentially as previously described [17] with minor modifications [11]. Early passage SCs were dissociated with trypsin to obtain a single cell suspension, which was seeded on top of a network of purified dissociated DRG neurons. SCs were allowed to repopulate the axonal outgrowth (proliferation phase) for 7–10 days in the absence of ascorbate or cAMP-stimulating agents before inducing myelination by the addition of L-ascorbic acid (50 μg/ml) and 5% FBS (myelination phase) alone or together with cAMP-stimulating agents, as indicated in the figure legends. In the co-culture system, SCs require a prolonged proliferation phase that enables repeated cycles of cell division prior to becoming competent to myelinate axons [45]. Medium changes were performed on a 3-day basis up until cell fixation and analysis (typically 12 days after initial cAMP or ascorbate supplementation). We have found that in the presence of high concentrations of cAMP-stimulating agents (e.g. 100 μM or higher) SCs consistently fail to express MBP or myelinate axons even in the presence of ascorbate. We suspect that high cAMP concentrations are not well tolerated by the DRG neurons, as we have seen evidence of neuronal/axonal loss with prolonged treatment (not shown). Because low concentrations of cAMP analogs allow for effective O1 and MBP expression while preserving the neurons, we have decided to use low concentrations of cAMP analogs (up to 20 μM) and forskolin (up to 0.5 μM) in all of our experiments involving SC-neuron cultures. SC-neuron cultures were analyzed for the expression of a variety of markers. Myelinating SCs were identified by their characteristic Krox-20 positive, O1 positive, MBP positive phenotype. Co-staining of co-cultures with collagen type IV or laminin antibodies was also performed to reveal the production of a basal lamina. Neuron-free SC cultures and SC-neuron cultures growing in the absence of ascorbate do not typically exhibit the expression of extracellular collagen type IV. Co-cultures were also routinely co-stained with antibodies against neurofilament (axonal marker) and DAPI (nuclear marker), which served as reference controls for the extension of the axonal web and the location of the cells, respectively. To serve as negative controls, we established non-myelinating SC-neuron cultures that were maintained in the absence of ascorbate supplementation throughout the time course of the experiments. SCs in these cultures typically lack the expression of markers of myelin (O1, MBP) and basal lamina (collagen type IV) regardless of their relative location within the axonal web. For reasons that are not well understood, SCs growing in the absence of neurons consistently die by apoptosis within 1 day after addition of ascorbate-containing medium (data not shown).

Quantification of O1 positive SCs in SC-neuron cultures was performed by automated fluorescence microscopy using a Thermo Scientific Cellomics ArrayScan VTI High Content System Reader, version 6.6.2.0 (High Content Screening Core Facility, The Miami Project to Cure Paralysis). Low magnification images (10x) of O1 (488), MBP (546), neurofilament (647) and DAPI (UV) staining were taken as serial images starting from the center toward the periphery of the well (24-well format). The number of O1 positive cells/condition was calculated in reference to the total number of cells (DAPI) using Target Activation Bioapplication software. At least 150 microscopic fields (about one fourth of the well’s surface) were routinely scanned for analysis. For O1 expression, data is represented as the number of fields containing more than fifty O1 positive SCs (out of 150 microscopic fields), as we found that this measure provides a reliable representation of the relative changes in O1 expression over large surface areas that exhibit spatial variability in the distribution of O1 positive cells. Quantitative analysis of the density of MBP positive cells was done by manual counting in selected images taken at a magnification of 10–20x. To emphasize the number and relative distribution of O1 positive SCs over a large culture area, results from myelination assays are presented as a composite of at least 20 consecutive low magnification fields. Selected areas are shown at a higher resolution to reveal features of the myelinating cells, such as their morphology and expression of MBP that cannot be appreciated at lower magnifications.

### Immunofluorescence microscopy

Cells were fixed with 4% paraformaldehyde followed by -20°C methanol. Cultures were blocked in 5% normal goat serum in PBS, incubated overnight at 4°C with the appropriate dilution of the primary antibody and then rinsed three times with PBS prior to incubation with Alexa-conjugated secondary antibodies. Labeling of cell surface O1 antigens was done by incubating living cells with hybridoma culture supernatant (20 min, RT) before paraformaldehyde fixation. An additional permeabilization step with a solution of 95% ethanol, 5% acetic acid was included for optimal detection of MBP. Cells were mounted with Vectashield containing DAPI (Vector Labs, Burlingame, CA) and analyzed by conventional or automated fluorescence microscopy using 10x to 40x magnification objectives. Conventional microscopy images were taken using a cooled digital black and white CCD camera (SensiCam QE, Cooke Corp.) coupled to an Olympus IX70 inverted fluorescence microscope. Images (8 bit, tiff format) were artificially colorized in RGB format, digitally processed and arranged for presentation using Adobe Photoshop V7.0 and Adobe Illustrator CS3. For cell quantification analysis, pictures from random fields were taken at 10–20x magnification and the number of cells labeled positive for the indicated markers was determined in reference to the total number of cells (DAPI staining). Cells were classified as positive or negative for the expression of Krox-20 (nuclear localization), O1 (membrane localization) and MBP (myelin segments) in comparison to non-treated controls and without regard to the variability of staining intensity shown by individual cells. At least 2,000 cells were analyzed in each sample.

### Western blots

Total cell and tissue lysates were prepared as reported previously [33]. Equal protein samples (typically 5–10 μg protein/lane) were subjected to electrophoresis using 8, 10 or 12% SDS-polyacrylamide gels, depending on the molecular weight of the protein of interest. Proteins were transferred to polyvinylidene fluoride membranes (Millipore, Bedford, MA) by a liquid transfer method. Membranes were blocked with ECL blocking agent (GE Healthcare) in Tris-Buffer saline containing 0.05% Tween-20 (TBS-T) and incubated overnight with a 1:1000 dilution of each primary antibody. The membranes were washed 3 times with TBS-T prior to incubation with horseradish peroxidase (HRP)-conjugated secondary antibodies (Promega). Immunoreactive protein bands were detected by enhanced chemiluminescence (ECL) using ECL Advanced or ECL Plus (GE Healthcare) depending on signal intensity. The expression of β-actin was used as a loading and reference control.

### Detection of PKA activity and cAMP

To determine the relative changes in the levels of PKA activity by western blot and immunofluorescence microscopy, we assessed the phosphorylation of specific PKA substrates using an antibody that recognizes PKA-specific phospho-motifs ([RR]-x-[S*/T*]) in target proteins (Cell Signaling Tech, Beverly, MA). For cell immunostaining, methanol-fixed cells were incubated (4 h, RT) with a 1:200 dilution of the P-PKA substrate antibody. For western blot, antibodies were used at 1:1000 (ON incubation, 4°C). We have shown previously that the P-PKA substrate antibody detects both cytoplasmic and nuclear phosphorylated PKA substrates in SCs and that the extent of P-PKA substrate immunoreactivity is directly proportional to the levels of PKA activation and intracellular cAMP, thus providing an accurate estimation of the relative levels of bioactive cAMP inside cells [33]. We previously confirmed the specificity of the P-PKA substrate signal by showing that PKA-selective rather than EPAC-selective analogs of cAMP elicit an increase in the P-PKA substrate signal [11, 33]. The detection of the relative changes in P-PKA substrate expression enables a direct comparison of immuno-staining and western blot results as well as the detection of regional differences such as immunoreactivity in individual cells and the subcellular distribution of the signal. Important changes in cAMP and PKA activity have also been observed in the DRG neurons (not shown), which makes it technically challenging to interpret results from standard evaluations of cAMP or PKA activity using whole cell lysates. Of note, pictures of immunofluorescence microscopy were taken under identical conditions of time of exposure and image acquisition parameters. Thus, the overall intensity of the P-PKA substrate signal in different images can be directly compared. The total levels of PKA catalytic subunit expression are shown as a reference control in western blot experiments.

The detection of the relative content and distribution of the cAMP signal in individual cells was determined by using a cAMP-specific antibody that immuno-reacts with cAMP in paraformaldehyde fixed cells. Immunostaining experiments for cAMP were performed according to the manufacturer’s instructions (Chemicon).

### Transmission electron microscopy (TEM)

SC-neuron cultures growing on PLL-laminin-coated glass cover slips were fixed overnight in 2% glutaraldehyde-100 mM sucrose and then rinsed in 0.15 M phosphate buffer before post-fixing for 1 h with 2% OsO4. Subsequently, cells were rinsed, dehydrated in graded ethanol solutions and embedded in Embed (Electron Microscopy Sciences, Hatfield, PA). Thin sections obtained with a Leica Ultracut E microtome were stained with uranylacetate/lead citrate for examination in a Philips CM-10 transmission electron microscope.

### Statistical Analysis

The statistical significance of the different treatments was analyzed relative to each control group by a one-way analysis of variance (ANOVA) follow by a post-hoc Tukey test using IBM SPSS statistics software. A p-value <0.05 was interpreted as statistically significant. Data from a representative experiment out of at least three independent experiments performed are shown. All experimental conditions were analyzed in triplicate samples.

## Supporting Information

S1 Fig Effect of high doses of CPT-cAMP on O1 and MBP expression in axon-associated SCs. SC-neuron cultures were treated and analyzed as described in Fig. 6 with the exception that a high concentration of CPT-cAMP (250 μM) was used for stimulation. The cultures were stained with O1 (green), MBP (red), and neurofilament (blue, upper panels) antibodies and counterstained with DAPI (blue, lower panels). Selected areas (white boxes) are shown at a higher magnification to reveal details of the morphology of the cells and their levels of expression of O1 (green) and MBP (red). Note that in the presence of high doses of CPT-cAMP, SCs massively express O1 but fail to myelinate, as judged by the inability of the cells to acquire an elongated bipolar phenotype and enhance their expression of MBP.
(TIF)
