# Supplementary figures and images for: Requirement of cAMP Signaling for Schwann Cell Differentiation Restricts the Onset of Myelination
Source: PLoS One. 2015 Feb 23;10(2):e0116948. doi: 10.1371/journal.pone.0116948 (PMC4338006; doi:10.1371/journal.pone.0116948)

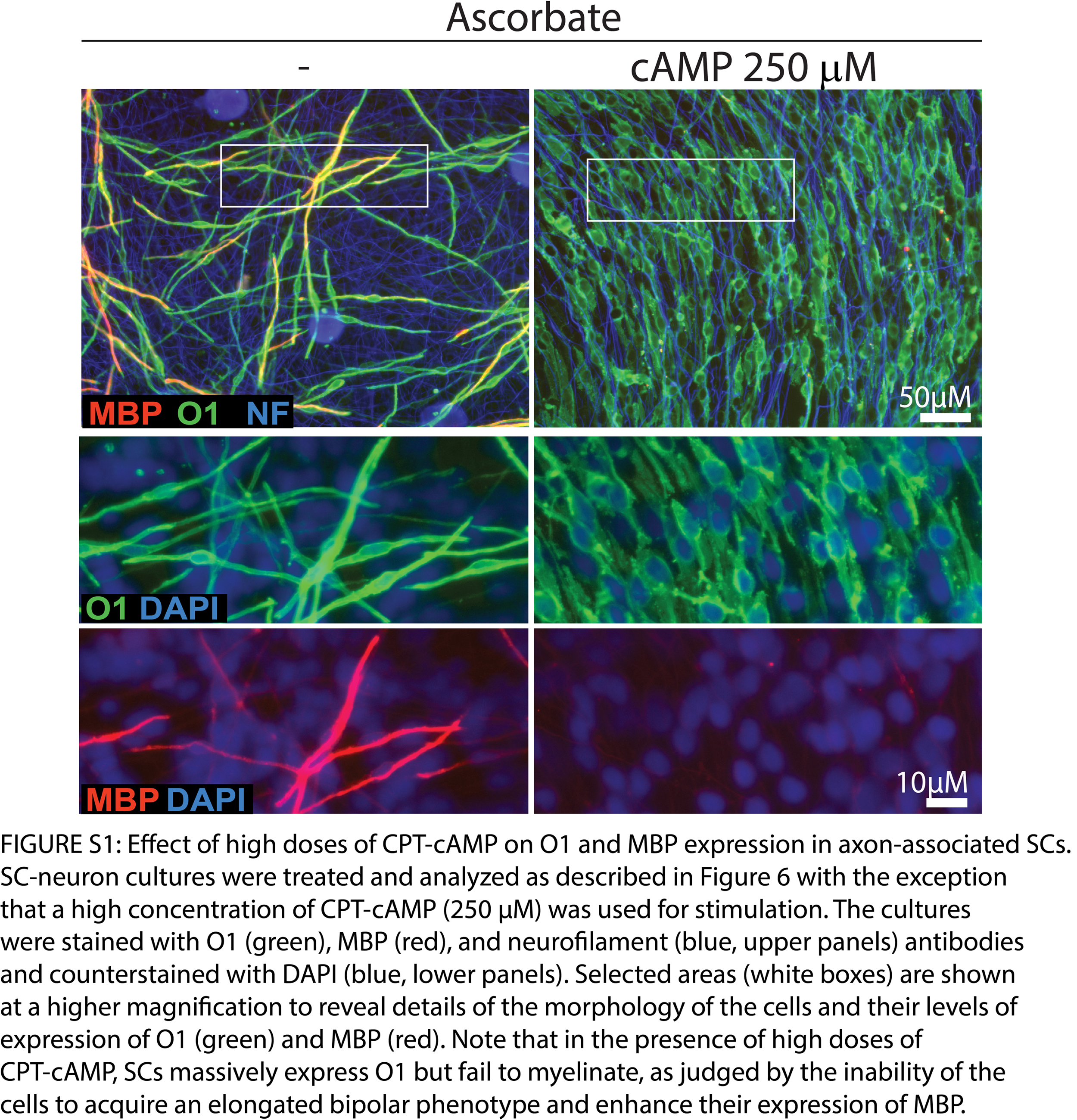

Supplement: S1 Fig — SC-neuron cultures were treated and analyzed as described in Fig. 6 with the exception that a high concentration of CPT-cAMP (250 μM) was used for stimulation. The cultures were stained with O1 (green), MBP (red), and neurofilament (blue, upper panels) antibodies and counterstained with DAPI (blue, lower panels). Selected areas (white boxes) are shown at a higher magnification to reveal details of the morphology of the cells and their levels of expression of O1 (green) and MBP (red). Note that in the presence of high doses of CPT-cAMP, SCs massively express O1 but fail to myelinate, as judged by the inability of the cells to acquire an elongated bipolar phenotype and enhance their expression of MBP. (TIF) [file pone.0116948.s001.tif]
